# The role of miRNAs in T helper cell development, activation, fate decisions and tumor immunity

**DOI:** 10.3389/fimmu.2023.1320305

**Published:** 2024-01-09

**Authors:** Shi-Jun Xu, Jin-Hua Chen, Suhwan Chang, Hai-Liang Li

**Affiliations:** ^1^ Department of Interventional Radiology, The Affiliated Cancer Hospital of Zhengzhou University, Henan Cancer Hospital, Zhengzhou, Henan, China; ^2^ Henan Medical Device Engineering Research Center of Interventional Therapy for Non-vascular Tumors, Henan Cancer Hospital, Zhengzhou, Henan, China; ^3^ Department of Pharmacy, The Affiliated Cancer Hospital of Zhengzhou University, Henan Cancer Hospital, Zhengzhou, Henan, China; ^4^ Department of Physiology, University of Ulsan College of Medicine, Asan Medical Center, Seoul, Republic of Korea; ^5^ Department of Radiology, The Affiliated Cancer Hospital of Zhengzhou University, Henan Cancer Hospital, Zhengzhou, Henan, China

**Keywords:** miRNAs; T helper cell, tumor microenvironment, development, activation, fate decisions, tumor immunity

## Abstract

T helper (Th) cells are central members of adaptive immunity and comprise the last line of defense against pathogen infection and malignant cell invasion by secreting specific cytokines. These cytokines then attract or induce the activation and differentiation of other immune cells, including antibody-producing B cells and cytotoxic CD8^+^ T cells. Therefore, the bidirectional communication between Th cells and tumor cells and their positioning within the tumor microenvironment (TME), especially the tumor immune microenvironment (TIME), sculpt the tumor immune landscape, which affects disease initiation and progression. The type, number, and condition of Th cells in the TME and TIME strongly affect tumor immunity, which is precisely regulated by key effectors, such as granzymes, perforins, cytokines, and chemokines. Moreover, microRNAs (miRNAs) have emerged as important regulators of Th cells. In this review, we discuss the role of miRNAs in regulating Th cell mediated adaptive immunity, focusing on the development, activation, fate decisions, and tumor immunity.

## 1 Introduction

CD4^+^ T helper (Th) cells are core members of the adaptive immune system that orchestrate context- and pathogen-specific responses by secreting specific cytokines (1). These cytokines then attract or induce the activation and differentiation of other immune cells, including antibody-producing B cells and cytotoxic CD8^+^ T cells (2). Th cells regulate cellular and humoral immune responses to a wide range of pathogens or cancer cells and determine the success of vaccines (3). The development of Th cells origins from hematopoietic stem cells (HSC) in the bone marrow, goes through T cell lineage commitment, β-selection, and positive/negative selection stages to generate single-positive CD4^+^ cells (4). In general, naïve CD4^+^ T cells are inactive and quiescent. However, upon antigen recognition and co-stimulation, CD4^+^ T cells are activated and switch from a resting state to clonal expansion. They differentiate into various effector T (Teff) cells to eliminate pathogens or tumorigenic cells, by activating different intracellular signal transduction cascades and master transcription factors. During this process, CD4^+^ T cells differentiate into Th cells, such as Th1, Th2, Th17, T follicular helper (Tfh) cells, and induced regulatory T (iTreg) cells (3). Finally, most of these Th cells die, while a small number survive to differentiate into memory T cells, which are distributed in the tissue and provide an anatomically pervasive network for immunosurveillance (5).

Owing to the continuous evolution of living organisms, tumor cells have gained the ability to escape immunosurveillance. The tumor microenvironment (TME), especially the tumor immune microenvironment (TIME), is the primary arena through which tumor cells can overcome the immune system (6, 7). There are many types of immune cells in the TIME, among which Th cells are the most abundant. Generally, when tumor cells develop, inflammatory activated immune cells infiltrate the TIME to clear the tumor cells; this is known as an immunologically “hot” tumor. However, sometimes tumor cells can re-educate immune cells in the TIME to switch off their immunosurveillance and prevent activated immune cells from entering the TIME; this is known as an immunologically “cold” tumor (7). Patients with “hot” tumors receive more benefits from clinical therapy than patients with “cold” tumors. Given the limited number of naïve T cells (7), the generation, activation and infiltration of activated Teff cells in the TIME are crucial.

Previous research has shown that granzymes, perforins, cytokines and chemokines are key regulators of Th cell development and activation (1), and recent studies, benefiting from the development of RNA-sequencing methods, demonstrated that transcription factors, post-transcriptional regulators, and microRNAs (miRNAs) are also important factors in Th cell fate decisions (2, 4, 8). miRNAs are small non-coding RNAs (~23nt) that participate in almost all biological processes including Th cell development, differentiation, migration, activation, and cell fate decisions (9). They generally bind to the 3’untranslated region (UTR) of target mRNAs to repress target gene translation at the post-transcriptional level (10). Because transcription factors, cytokines and chemokines are also regulated by miRNAs, they are essential players in Th cell activation and function. In this review, we highlight the miRNAs that play vital roles in Th cell development, differentiation, activation, and fate decisions, as well as tumor immunity.

## 2 miRNAs involved in Th cell development and activation

### 2.1 miRNAs in Th cell development

**Figure 1:**
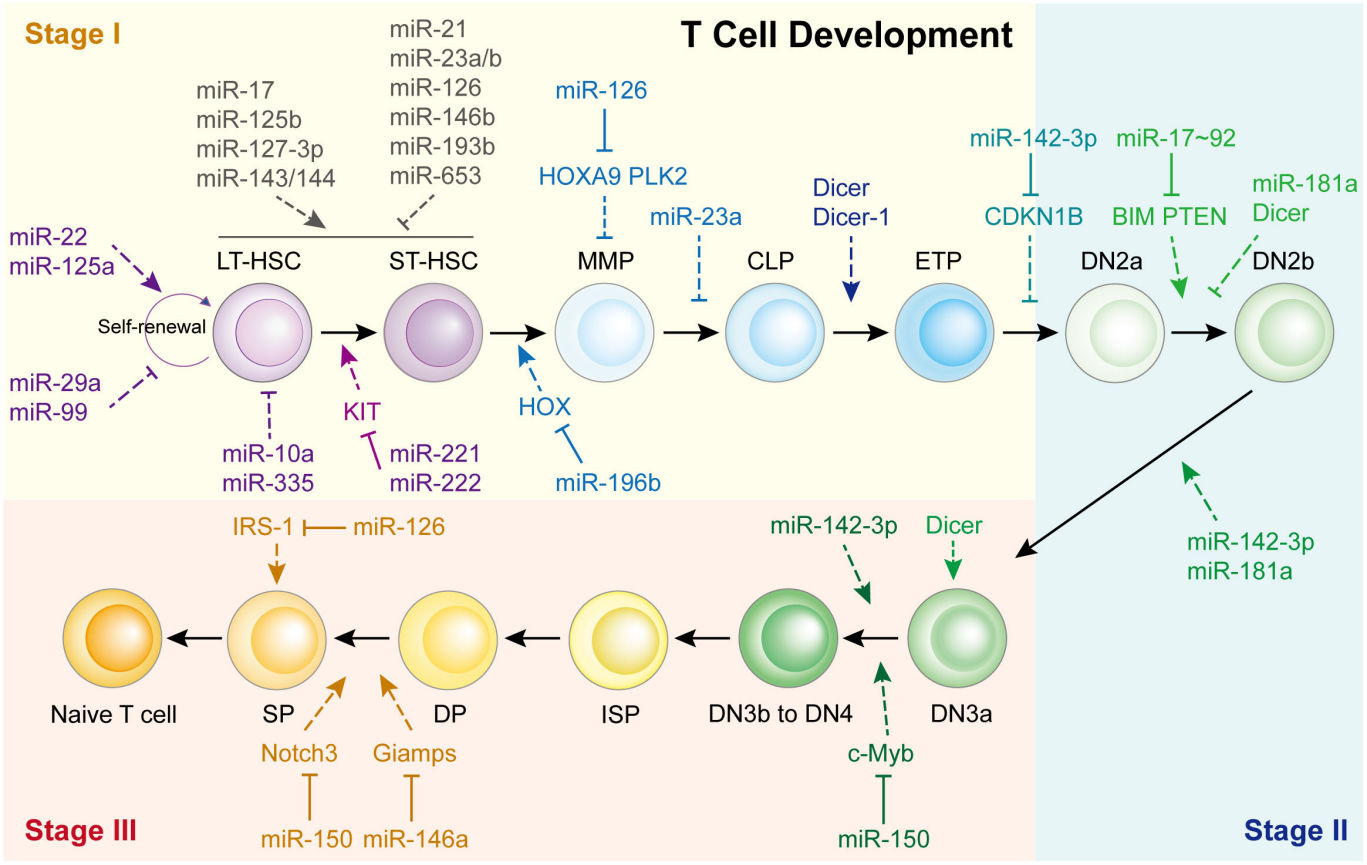
miRNAs in Th cell development. HSC, hematopoietic stem cells; LT-HSC, long-term HSC; ST-HSC, short-term HSC; MPP, multipotent progenitor; CMP, common myeloid progenitor; CLP, common lymphoid progenitor; ETP, early T cell progenitor; DN, double-negative; DP, double-positive.

The thymus is a unique organ involved in T-cell lineage commitment. Based on the expression of co-receptors (CD4 and CD8) and markers (CD25, CD28 and CD44), the development and differentiation of T cells into CD4^+^ Teff cells or CD8^+^ cytotoxic T cells can be mainly divided into three stages: the T-cell lineage commitment, β-selection, and positive/negative selection stages (Stages I-III, respectively) (4, 11, 12). In Stage I, T cells are originally derived from HSCs located in the bone marrow. They progress from multi-potent progenitors (MPPs), into common lymphoid progenitors (CLPs), and are recruited to the thymus (13). These CLPs then differentiate into early T-cell progenitors (ETPs) and double-negative 2a (DN2a) cells via the promotion of Notch signaling within the thymic microenvironment (4, 14). In Stage II, the DN2a cells undergo γ, δ and β T cell receptor (TCR) gene rearrangement, developing into DN2b and DN3a cells. In Stage III, the T cell lineages complete their commitment through TCRα rearrangement to generate double-positive (DP) cells, producing CD4^+^ or CD8^+^ T cells via negative or positive selection. This process begins with DN3b or DN4 cell formation, which gradually evolves into immature single-positive (ISP) cells, DP cells, and finally develop into single-positive CD4^+^ or CD8^+^ T cells. Although various studies have demonstrated that activated Notch signaling and its associated transcription factors are vital during T cell development (4), recent findings have shown that miRNAs also play essential roles in T cell development (**Figure 1**).

The first study was reported in 2005, Muljo et al. revealed that Dicer-1 deletion in the mouse thymus reduces the number of CD4^+^ T cells and aberrant Th cells (15). While Cobb et al. indicated that deletion of Dicer at an early stage of T cell development reduced the survival of TCR α/β lineage cells but did not affect TCR γ/δ thymocytes, implying that Dicer might be dispensable for CD4/8 lineage commitment (16).

Consistent with these reports, it appears that each T cell development step is affected by miRNAs, especially the beginning of Stage I (**Figure 1**). For example, miR-22 (17), miR-125a (18), miR-29a (19), and miR-99 (20) control HSC renewal. Eleven miRNAs functioned together to manipulate HSC homeostasis and expansion. miR-10a and miR-335 weaken long-term HSCs (21), whereas miR-221/222 repress the differentiation of long-term HSCs into short-term HSCs by targeting the KIT receptor (22). In short-term HSCs that transform into multipotent progenitors, miR-196b is the most abundant miRNA and regulated by the HSC transcription factor family mixed lineage leukemia. It modulates HSC homeostasis and lineage commitment possibly by directly targeting homeobox (HOX) (13). Li et al. showed that miR-126 inhibits apoptosis and increases the viability of acute myeloid leukemia cells and enhanced the colony-forming ability of mouse normal bone marrow progenitor cells alone by targeting tumor suppressor polo like kinase 2, which facilitates the development of leukemia (23). While, Shen et al. found that miR-126 is expressed in hematopoietic stem cells and regulates normal hematopoietic cells function by targeting HOXA9, which is validated by using specific bone marrow-derived cell lines (24).

However, only a limited number of miRNAs have been found to regulate Stage II and Stage III (**Figure 1**). The most representative miRNA is miR-142-3p, which is essential for normal thymic cellularity and peripheral T cells. Mechanistically, it accelerates early T-cell progenitor proliferation from T lineage progenitors to DN4 cells by targeting cyclin-dependent kinase inhibitor 1B and other critical target genes (25). Another important one is miR-150, which is up-regulated during normal T-cell maturation, however, it blocks T cell development. Ghisi et al. revealed that it reduces Notch3 levels in T-cell lines and represses the SP-DP step, thus damaging T-cell development and physiology (26). While, Xiao et al. indicated that c-Myb, a transcription factor controlling multiple steps of lymphocyte development, is targeted by miR-150 (27). Further study showed that miR-150 controls c-Myb expression in a dose-dependent manner, which dramatically affects lymphocyte development and response at the DN3 to DN4 transition. Other miRNAs participated in T cell development are shown in **Figure 1**; **Supplementary Table 1**.

### 2.2 miRNAs in Th cell activation and proliferation

Generally, naïve CD4^+^ T cell activation and differentiation begin with antigen recognition. In the thymus, TCR, located on the surface of naïve CD4^+^ T cells, binds to and recognizes pathogen-derived peptides. These, in turn, produce inositol-triphosphate and diacylglycerol via phospholipase C, which cleaves phosphatidyl-inositol 4,5-bisphosphate (PIP2) (2). Inositol-triphosphate regulates Ca^2+^ influx to activate the transcription factor nuclear factor of activated T cells (NFAT), while diacylglycerol activates AP1 and NF-κB, leading to the activation of T cells and expression of interleukin (IL-2), the hallmark cytokine of activated T cells (28, 29). During the co-stimulation step, antigen-presenting cells express B7 family members that bind to the CD28 receptor on T cells and activate class I phosphatidyl-inositol 3-kinase (PI3K) to produce phosphatidyl-inositol 3,4,5-triphosphate (PIP3) from PIP2 (2). PIP3 signaling activates many transcription factors, such as NF-κB and mTOR, which promote cell survival, enhance transduction from TCR, and increase cellular metabolic activity. Moreover, phosphatase and tensin homologs (PTEN) negatively regulate PI3K/AKT pathway activation, as they dephosphorylate PIP3 to generate PIP2 (2, 30).

During these two processes, miRNAs involved are limited (**Figure 1**). Of note, the miR-17-92 cluster is the classical miRNA cluster regulating the PI3K/AKT/mTOR pathway. It directly binds to the PTEN 3’ UTR and restrains PTEN expression, which enhances AKT-mTOR signaling, promotes Tfh and Th17 cell differentiation, and reduces iTreg differentiation (31). miR-19b facilitates Th1 differentiation and interferon (IFN)-γ production by directly targeting PTEN (32). miR-17 also increases IFN-γ expression and promotes Th1 cell response by targeting transforming growth factor beta (TGF-β) receptor 2 and cyclic antimicrobial peptide responsive element binding protein (CREB). Because of its vigorous control over the Th1/Treg balance, the loss of miR-17-92 in CD4 T cells results in tumor evasion (32). In addition to the miR-17-92 cluster, miR-21 facilitates CD4^+^ T cell polarization toward Th2 cells by regulating the PTEN/PI3K/AKT pathway, which accelerates arsenite-induced hepatic fibrosis (33). miR-99a (34), miR-150 (34), and miR-183 (35) directly bind to mTOR mRNA and suppress its expression, which attenuates Th1 and Th17 cells differentiation, but promotes Treg differentiation. Of note, highly expressed miR-150 contributes at physiological levels while the lower expressed miR-99a is strongly induced by Treg cell inducer. These two miRNAs cooperate together to promote Treg but impair Th17 induction (34).

Once activated by antigens, Th cells switch from the resting state to clonal expansion. This switch requires the inactivation of the transcription factor Foxo1, which is expressed in resting Th cells, to suppress cell proliferation (36). During this process, miR-182 is induced by IL-2 and, in turn, targets Foxo1 to promote the clone expansion of activated Th1, Th2, and Th17 cells, indicating that miR-182 plays vital roles in the physiological regulation of IL-2-driven Th cell-mediated immune responses (36). A separate study demonstrated that miR-92a directly targets Foxo1 to restrict Treg induction and supports the Th17 response. Through sustaining the imbalance of Th17/Treg cells, miR-92a promotes CNS autoimmunity (37). miR-425 and miR-873 also target Foxo1 to facilitate the differentiation of naïve CD4^+^ T cells into Th17 cells in inflammatory bowel disease and the pathogenesis of systemic lupus erythematosus, respectively (38, 39). Moreover, miR-125b, which is highly expressed in human naïve CD4^+^ T cells, inhibits T cell differentiation by targeting IFN-γ, IL-2 receptor subunit beta, IL-10 receptor subunit alpha, and PR/SET domain 1 (40). miR-146a also negatively regulates T cell activation by inhibiting NF-κB signaling (41) (**Figure 1**).

## 3 miRNAs in Th cell differentiation and fate decision

The cytokine microenvironment affects Th cells in different ways. Activation of different intracellular signal transduction cascades and master transcription factors shapes the differentiation and plasticity of Th cells. This leads to the maturation of five major cell subsets with different functions, classified based on the cytokines they secrete: Th1, Th2, Tfh, Th17, and iTreg (2, 3). Here, we discuss the role of miRNAs in Th cell differentiation and cell fate decisions, focusing on master transcription factors and related intracellular signal transduction cascades.

### 3.1 miRNAs in Th1 cell differentiation and fate decision

Th1 cells regulate immune responses against viruses, intracellular pathogens and tumors via predominantly producing IFN-γ (3). Th1 cell dysfunction induces autoimmune diseases. Upon IL-12/signal transducer and activator of transcription (STAT) 4 and IFN-γ/STAT1 signaling activation, phosphorylated STAT4 and STAT1 bind to the T-bet promoter and accelerate T-bet (a master regulator of Th1 cell) and IFN-γ expression (42). CD4^+^ T cell then differentiates into Th1 cell, which promotes macrophage and cytotoxic T cell activation and function as pro-inflammatory Teff cell (2).

**Figure 2:**
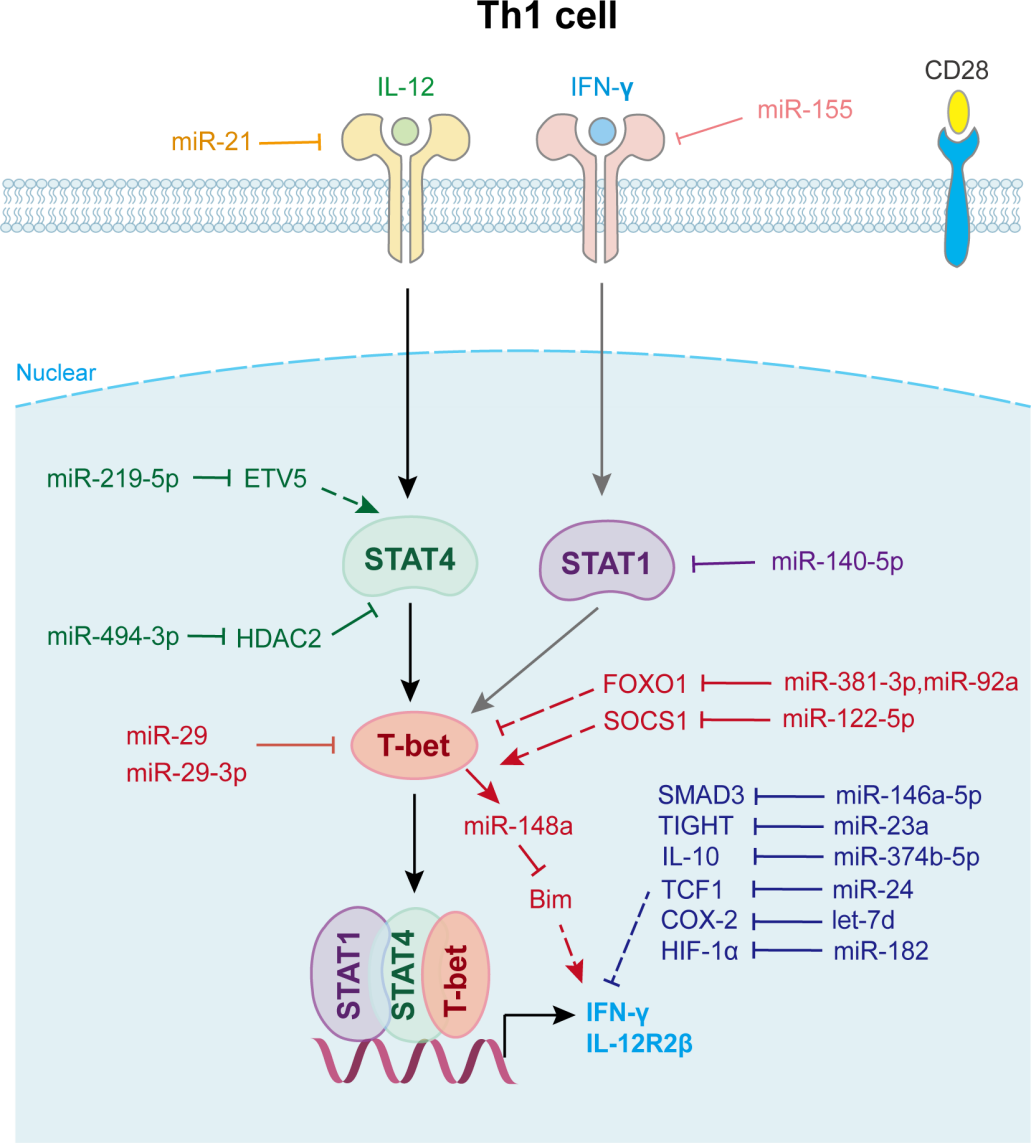
miRNAs in Th1 cell differentiation and fate decision. IL-12, interleukin-12; IFN-γ, interferon-gamma; STAT1 or STAT4, signal transducer and activator of transcription 1 or 4; T-bet, T-Box expressed in T cells; IL-12R2β, interleukin-12 receptor 2 beta.

In Dicer-deficient CD4^+^ T cells, Muljo et al. found increased differentiation into effector cells expressing T-bet and IFN-γ (15). Later research showed that T cells lacking Drosha or DGCR8 have a phenotype similar to that of aberrant Th1 cell differentiation (43). Several miRNAs are involved in Th1 cell differentiation (**Figure 2**). For example, the overexpression of miR-29a and miR-29b reduced aberrantly high IFN-γ and T-bet levels (44). Further study indicated that miR-29 not only directly targets IFN-γ mRNA to control innate and adaptive immune responses to intracellular bacterial infection (45), but also targets T-bet and Eomesodermin (Eomes) (44) to reduce IFN-γ expression, thus limiting Th1 cell differentiation. miR-146a regulates Th1 cell differentiation, either by inhibiting IFN-γ expression or targeting STAT1 (46).

Another well-characterized immune cell-expressed miRNA, miR-155, is strongly up-regulated during T cell activation. It contributes to Th1 differentiation in CD4^+^ T cells by targeting the IFN-γ receptor alpha-chain, thus inhibiting IFN-γ signaling (47). Guan et al. revealed that the overexpression of miR-140-5p in peripheral blood mononuclear cells suppressed encephalitogenic Th1 cell differentiation by inhibiting STAT1 and downstream T-bet expression, which attenuates the progression of multiple sclerosis (48). Moreover, miR-24 drives the production of IFN-γ and IL-17 in T cells at least in part through targeting TCF1, a transcription factor known for its role in limiting Th1 and Th17 immunity (49). miR-210 induces Th17 and Th1 cell differentiation but inhibits Th2 differentiation by repressing STAT6 and LYN expression, thus promotes psoriasis-like inflammation (50). Activated Th1 cell also expresses elevated levels of certain miRNAs that promote their survival. miR-148a, which is specifically upregulated by Twist1 and T-bet in Th1 cell, is essential for Th1 cell survival as it targets proapoptotic gene Bcl2-interacting protein (Bim) expression (51) (**Figure 2**).

Moreover, Th1 cell differentiation can be indirectly affected by other immune cells via miRNAs (**Figure 2**). For example, miR-21 is highly expressed in dendritic cells (DCs) and directly targets and reduces IL-12p35 expression in DCs. Lower secreted IL-12 restrains T-bet and IFN-γ expression in CD4^+^ T cells, which represses the proliferation and survival of Th1 cells (52). Wu et al. found that miR-10a is decreased in the inflamed mucosa of inflammatory bowel disease patients, and downregulates mucosal inflammatory response. Further study showed that DC-derived miR-10a blocks Th1/Th17 cell immune responses and facilitates pathogenesis and progression of inflammatory bowel disease by suppressing IL-12/IL-23p40, subunits of IL-12 (53).

In addition, exosomes and other small extracellular vesicles also play important roles in Th1 cell differentiation (**Figure 2**). Zhu et al. found that miR-29a-3p contained in exosomes derived from granulocyte-like myeloid-derived suppressor cells restrains Th1 cell differentiation by targeting T-bet, which attenuates collagen-induced arthritis (54). Okoye et al. reported that Treg cell derived exosomes transfer let-7d to Th1 cell and suppresses its proliferation and cytokine secretion, which contributes to the suppression and prevention of systemic disease (55). Jiang et al. found that keratinocytes secrete small extracellular vesicles containing miR-381-3p. This miRNA targets Foxo1 and activates T-bet transcription to promote Th1 cell polarization and differentiation, which accelerates psoriasis development (56). Other miRNAs involved in Th1 cell fate decision are shown in **Supplementary Table 2**.

### 3.2 miRNAs in Th2 cell differentiation and fate decision

Th2 cells regulate immune responses against extracellular parasites including helminths, and dysfunctional Th2 cell responses can lead to allergies and asthma (3). Upon the activation of IL-4/STAT6 signaling, phosphorylated STAT6 activates GTAT3 transcription in collaboration with IL-2/STAT5. Activated STAT5, STAT6, and GTAT3 bind to the IL-4 promoter to facilitate its expression (57). Other transcription factors also affect Th2 cell differentiation. c-Maf cooperates with JunB to increase IL-4 transcription in Th2 cells (2). IFN-regulatory factor 4 (IRF4) is essential for Th2 cell development (58), and RUNX3 interacts with and attenuates GATA3 expression to restrain Th2 cell differentiation (59).

**Figure 3:**
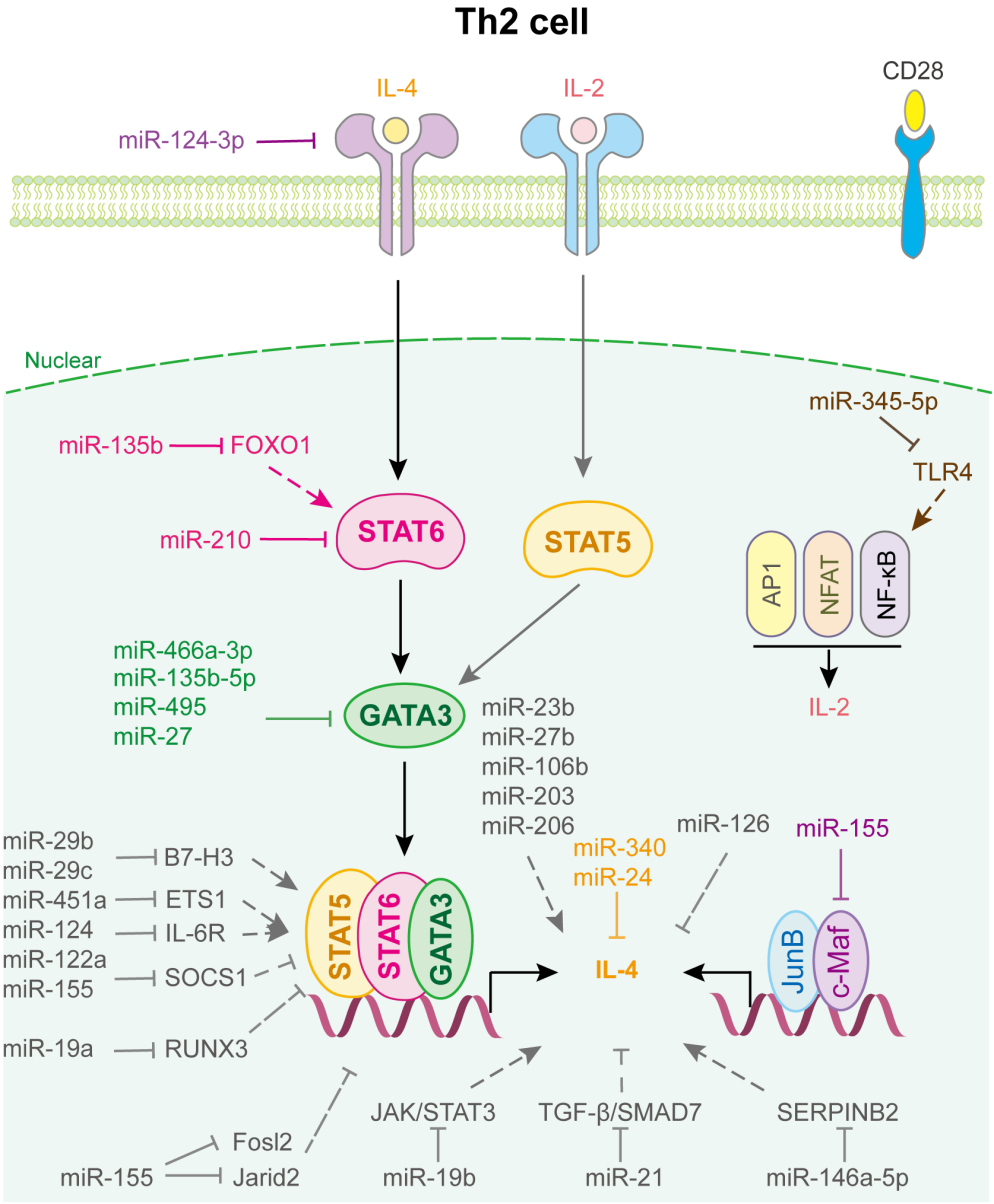
miRNAs in Th2 cell differentiation and fate decision. IL-4 or IL-2, interleukin-4 or 2; STAT5 or STAT6, signal transducer and activator of transcription 5 or 6; GATA-3, GATA binding protein 3; JunB, JunB proto-oncogene, AP-1 transcription factor subunit; c-Maf, proto-oncogene c-Maf; AP1, Jun proto-oncogene; NFAT, nuclear factor of activated T-cells; NF-κB, nuclear factor kappa B subunit 1.

miRNAs regulated Th2 cell differentiation and fate decision are displayed in **Figure 3**. miR-24 and miR-27 collaboratively control Th2 immunity to suppress allergic inflammation by targeting IL-4 and GATA3, respectively (60). miR-124-3p alleviates type 2 inflammatory response in allergic rhinitis via IL-4Rα (61). miR-340, down-regulated in CD226 deficient Treg, inhibits Th2 cell differentiation and cytokine production by directly targeting IL-4, which aggravates renal fibrosis (62). miR-466a-3p, miR-135b-5p and miR-495 target GATA3 to attenuate Th2 function in allergic rhinitis (63–65). Liu et al. found that miR-345-5p binds to the 3’UTR of toll-like receptor 4 (TLR4), reducing its expression and downstream NF-κB pathway activation, which decreases Th2 cell accumulation (66). In addition, T cell-intrinsic miR-155 decreases c-Maf, suppressor of cytokine signaling 1 (SCOS1), FOS-like 2 (Fosl2), and Jumonji- and AT-rich interaction domain containing protein 2 (Jarid2) to promote Th2 and Th17-biased responses in acute and chronic airway inflammation (67), whereas miR-19b suppresses JAK/STAT3 signaling to restrain Th2 cells function (68). Similarly, miR-21 affects the TGF-β/SMAD7 pathway to control Th2 cell differentiation (69).

Notably, miRNAs expressed in other immune cells also play important roles in regulating Th2 cell fate (**Figure 3**). For example, human mesenchymal stem cell-derived exosomes inhibited Th2 cell differentiation by regulating the miR-146a-5p/SERPINB2 pathway in patients with allergic rhinitis (70). Using loss- and gain-of-function strategies, Sun et al. found that miR-29c, which is expressed in macrophages of asthmatic children, regulates Th2 cell differentiation by directly targeting the co-stimulatory molecule B7-H3 (71). Coincidentally, Gu et al. indicated that miR-29b, also decreased in asthmatic children and expressed in macrophages, attenuates T cell differentiation into Th2 cell in asthma by targeting B7-H3 and STAT3 (72). Other miRNAs involved in Th2 cell fate are shown in **Supplementary Table 3**.

### 3.3 miRNAs in Th17 cell differentiation and fate decision

**Figure 4:**
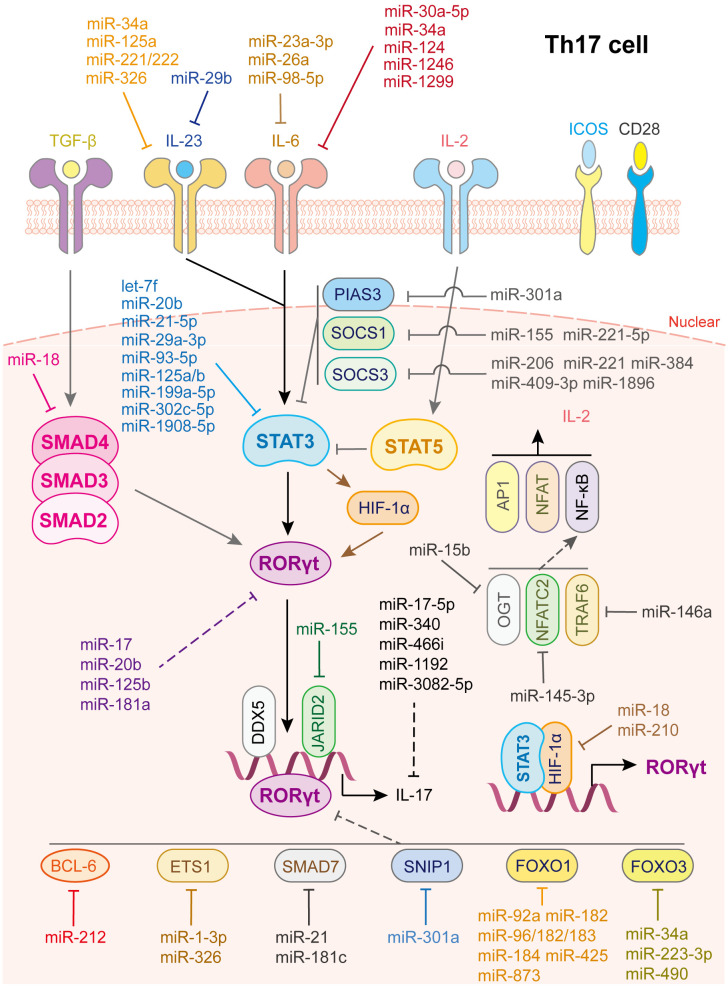
miRNAs in Th17 cell differentiation and fate decision. IL-23 or IL-6 or IL-2, interleukin- 23 or 6 or 2; TGF-β, transforming growth factor beta 1; STAT3 or STAT5, signal transducer and activator of transcription 3 or 5; RORγt, retinoid-related orphan receptor-gamma; SMAD2 or SMAD3 or SMAD4, SMAD Family Member 2 or 3 or 4; HIF-1α, hypoxia inducible factor 1 subunit alpha; OGT, O-linked N-Acetylglucosamine (GlcNAc) transferase; NFATC2, nuclear factor of activated T-cells 2; TRAF6, TNF receptor associated factor 6; AP1, Jun proto-oncogene; NFAT, nuclear factor of activated T-cells; NF-κB, nuclear factor kappa B subunit 1.

Th17 cells play vital roles in defense against bacteria and fungi, and their dysfunction is related to autoimmune diseases, including psoriasis, multiple sclerosis, colitis, arthritis, and asthma. Th17 cell is characterized by the expression of transcription factor retinoic acid receptor-related orphan receptor-γt (RORγt) and cytokines of the IL-17 family, such as IL-17A, IL-17F, IL-21, IL-22, and IL-26 (73, 74). The differentiation of Th17 cell is initiated by the activation of the IL-6/STAT3 pathway, which generates RORγt. TGF-β signaling and IL-21- and IL-23-mediated JAK/STAT3 signaling also participate in this step (2). Besides, IL-2/STAT5 signaling serves as a negative regulatory process via inhibiting IL-6/STAT3 activation. Many factors are involved in the regulation of Th17 cell fate. For example, DEAD-Box helicase 5 (75) and Jarid2 (76) are cofactors of RORγt. HIF-1α, directly activated by STAT3, collaborates with STAT3 to produce RORγt and IL-17 (77). TNF receptor associated factor 6 (TRAF6), NFAT cells 2 (NFATC2) and O-linked N-acetylglucosamine transferase (OGT), are positive regulators of the NF-κB pathway (78). These factors promote the differentiation of Th17 cell, whereas SMAD7, protein inhibitor of activated STAT 3 (PIAS3) and Smad nuclear interacting protein 1(SNIP1) (78), BCL-6 (79), ETS proto-oncogene 1 (ETS-1) (80), FOXO1 and FOXO3 (78) are negative regulators of RORγt and inhibit Th17 cell differentiation (**Figure 4**).

As for IL-6/JAK/STAT3 signaling activation, various miRNAs restrain STAT3 or RORγt activation by directly targeting key gene expression (**Figure 4**). IL-6 is a direct target of miR-23a-3p (81), miR-26a (82) and miR-98-5p (83), which modulates the inflammatory responses in aplastic anemia, multiple sclerosis, and gastric cancer, respectively. IL-6R or GP130 is the target of miR-30a-5p (84), miR-34a (85), miR-124 (86), miR-1299 (84) and miR-1246 (87). In this way, miR-34a serves as safeguard for Citrobacter-induced inflammatory colon oncogenesis (85), miR-124 alleviates collagen-induced arthritis (86), and miR-1246 attenuates hepatic ischemia reperfusion injury (87). IL-21R is the target of miR-30a (88) while IL-23R or IL-23 is the target of miR-29b (89), miR-125a (90), miR-34a (85), miR-326 (91) and miR-221/222 (92). Additionally, let-7f-5p (93), miR-29a-3p (94), miR-21-5p (94), miR-93-5p (54), miR-20b (95), miR-199a-5p (96), miR-125a/b (97), miR-302c-5p (98) and miR-1908-5p (99) directly target STAT3 to inhibit Th17 cells differentiation and activation. Notably, through repressing the function of Th17 cells or inducing Th17/Treg imbalance, let-7f-5p contributes to multiple sclerosis (93), miR-29a-3p and miR-21-5p, released from tumor-associated macrophages, promote the progression of epithelial ovarian cancer (94), miR-20b facilitates the pathogenesis of experimental autoimmune encephalomyelitis (95), miR-199a-5p alleviates immune thrombocytopenia (96), miR-125a/b attenuates colitis (97), miR-302c-5p weakens ulcerative colitis (98), and miR-1908-5p alleviates rheumatoid arthritis in mice (99).

In contrast, some transcription factors play vital roles in maintaining STAT3 signaling homeostasis, such as SOCS1, SOCS3 and PIAS3 (100, 101) (**Figure 4**). To date, miR-155 (102) and miR-221-5p (103) have been shown to directly target SOCS1, miR-206 (104), miR-409-3p, miR-1896 (105), miR-221 (106) and miR-384 (107) have been shown to target SOCS3, and miR-301a has been shown to inhibit PIAS3 expression (108) to limit STAT3 activation and facilitate Th17 cell differentiation. In addition, some miRNAs directly target and reduce RORγt and IL-17 expression; miR-181a (109), miR-17 (110), miR-20b (95), and miR-125b (111) decrease RORγt expression while miR-17-5p (112), miR-340 (113), miR-466i (114), miR-1192 (115), and miR-3082-5p (116) inhibit IL-17 or IL-17A expression.

Moreover, miRNAs regulate co-factor expression, thus affecting Th17 cell activation (**Figure 4**). miR-92a (37), miR-182, miR-183-96-182cluster (117), miR-184 (118), miR-425 (38), and miR-873 (39) facilitate Th17 cell differentiation by directly inhibiting Foxo1 expression while miR-34a (119), miR-223-3p (120), and miR-490 (115) promote Th17 cell differentiation by directly inhibiting Foxo3 expression. In addition, miR-210 (121) and miR-18 (122) target HIF-1α thereby inhibiting RORγt activation. miR-1-3p (123) and miR-326 (80) promote Th17 cell differentiation by directly inhibiting ETS-1 expression. miR-145-3p reduces NFATC2 expression (124), miR-146a suppresses TRAF6 expression (125) and miR-15b inhibits OGT expression (126), contributing to Th17 cell activation. As for TGF-β signaling, miR-18 targets SMAD4 (122) to suppress Th17 cell differentiation whereas miR-21 (69) and miR-181c (127) target SMAD7 to facilitate Th17 cell differentiation. miR-212 promotes IL-17-producing T-helper cell differentiation by targeting BCL-6, a negative regulator of Th17 cell differentiation (79). Last but not least, elevated miR-301a in peripheral blood mononuclear cells and inflamed mucosa of patients with inflammatory bowel disease facilitates Th17 cell differentiation by reducing SNIP1 expression (128).

In addition, Th17 cell differentiation and activation are influenced by other immune cells, such as DCs and macrophages (**Figure 4**). miR-29, the only up-regulated miRNA in mature DCs and significantly downregulated in tumor-associated DCs, downregulates IL-23 and antagonizes the Th17 inflammatory response. Further study showed that multiple myeloma reprograms DCs by reducing miR-29b, which facilitates multiple myeloma cell growth and survival (89). In contrast, transfection of miR-10a into human monocyte-derived dendritic cells led to a decrease in IL-12/IL-23p40 and markedly suppressed Th1 and Th17 cell responses in inflammatory bowel disease (53). Myeloid-derived suppressor cells (MDSC) not only promote Th17 cell differentiation or polarization through the arginase-1/miR-322-5p/TGF-β pathway (129), but also via the miR-542-5p/TGF-β/Smad3 pathway (130), to accelerate systemic lupus erythematosus progression. B7-H3 is the direct target of miR-29c and the transfection of anti-miR-29c into macrophages enhances RORγt and GATA3 expression in co-cultured CD4^+^ T cells. This regulation, in turn, elevates IL-4 and IL-17 levels, facilitates Th17 cell responses, which provide a new target for treatment of children with allergic asthma (71). Other miRNAs involved in Th17 cell differentiation and activation are summarized in **Supplementary Table 4**.

### 3.4 miRNAs in Tfh cell differentiation and fate decision

Tfh cells help B cells differentiate into long-lived antibody-secreting plasma cells or memory B-cells via co-localization and interaction with B cells. This is crucial for controlling the development of humoral immunity, the generation of specific high-affinity antibodies, and the maintenance of long-term protective immunity (2, 131). Tfh cells are characterized by expression of the master transcription factor Bcl-6, inhibitory receptor programmed cell death protein 1, inducible T-cell costimulatory (ICOS), CXC-chemokine receptor type 5 (CXCR5), CXC-chemokine ligand 13 (CXCL13), and IL-21, which are important for the migration of Tfh cells toward B-cell follicles (132). Upon activation of IL-6/STAT3 signaling, Bcl-6 expression is triggered. IL-21 promotes this process, whereas IL-2/STAT5 signaling acts as a brake to antagonize this process by activating Blimp1, inhibiting Bcl-6 expression (133).

**Figure 5:**
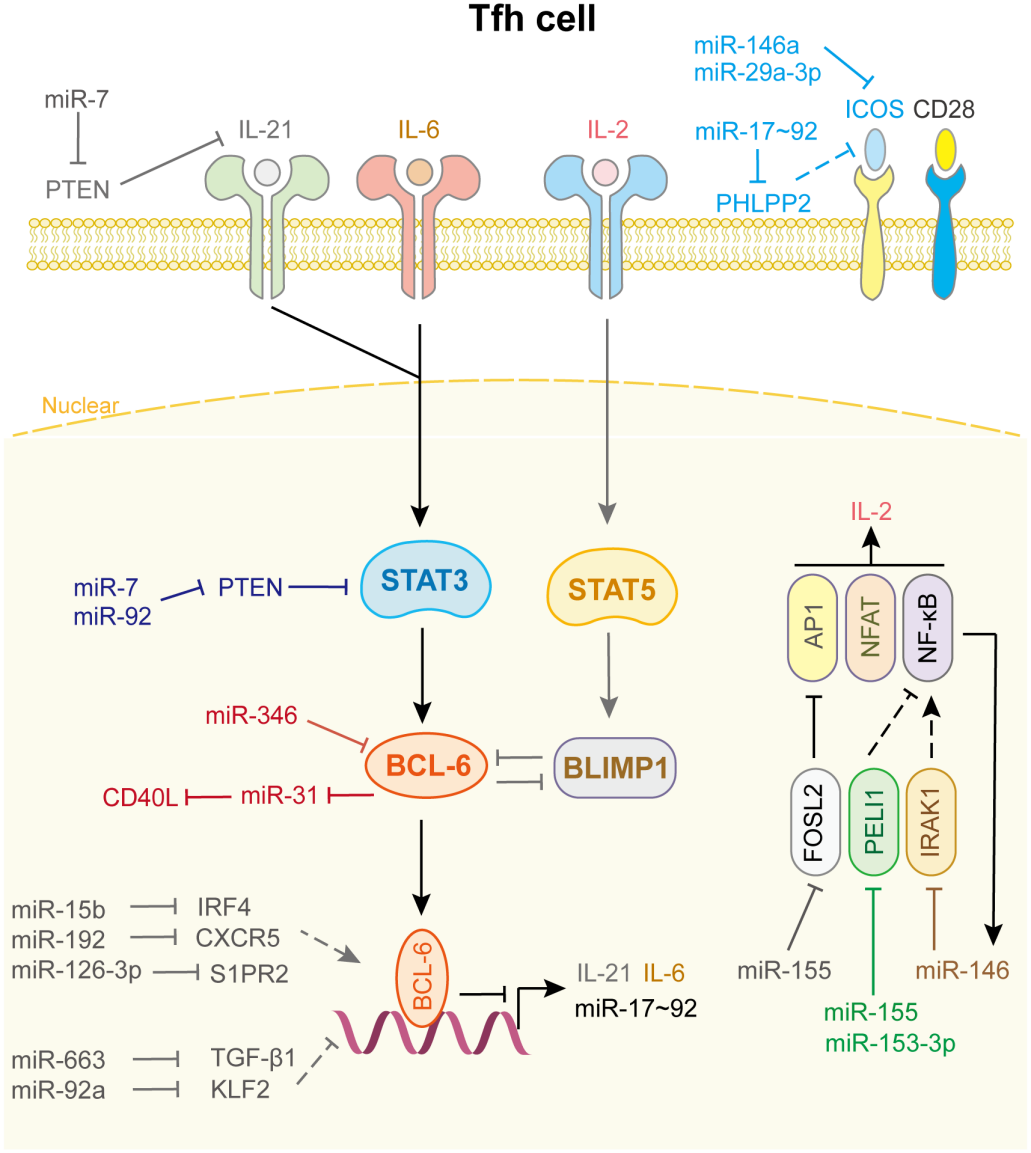
miRNAs in Tfh cell differentiation and fate decision. IL-21 or IL-6 or IL-2, interleukin-21 or 6 or 2; ICOS, inducible T cell co-stimulator; STAT3 or STAT5, signal transducer and activator of transcription 3 or 5; BCL-6, B-cell lymphoma 6 protein; BLIMP1, B-lymphocyte-induced maturation protein 1; FOSL2, FOS like 2, AP-1 transcription factor subunit; PELI1, pellino E3 ubiquitin protein ligase 1; IRAK1, interleukin 1 receptor associated kinase 1; AP1, Jun proto-oncogene; NFAT, nuclear factor of activated T-cells; NF-κB, nuclear factor kappa B subunit 1.

As shown in **Figure 5**, only a few miRNAs participate in the differentiation and fate decision of Tfh cell. Among these miRNAs, the most common are the miR-17-92 family, miR-155, and miR-146a. Kang et al. reported that the deletion of the miR-17-92 family in T cells results in defects in Tfh cell differentiation, germinal center formation, and antibody responses. Further investigation indicated that this family directly targets the phosphatase PH domain and leucine-rich repeat protein phosphatase 2 to obstruct the migration of CD4^+^ T cells into B-cell follicles (134). In addition, this cluster inhibits PTEN and the proapoptotic protein Bim, which promotes the accumulation of antigen-experienced T cells and germinal center B cells. And lymphocytes with high miR-17-92 level show more proliferation and less activation-induced cell death, which leads to lymphoproliferative disease and autoimmunity in mice (135). miR-29a-3p (136) and miR-146a (137) directly binds to Icos mRNA and suppresses its translation, thus hampering the co-stimulation process and Tfh cell differentiation and accumulation. Interestingly, in miR-146a^-/-^ mice, Tfh cells accumulate and promote an inflammation response when miR-155 is overexpressed, indicating that miR-155 drives Tfh cell differentiation and fate decision. Mechanistically, miR-155 repressed Fosl2 expression, which negatively regulated Tfh cell differentiation by inhibiting AP-1 expression (138). Liu et al. uncovered another mechanism by which miR-155 regulates Tfh cell fate decision by targeting Pellino E3 ubiquitin protein ligase 1 (Peli1). Peli1 is a ubiquitin ligase that promotes the degradation of the NF-κB family transcription factor c-Rel, which controls Tfh cell proliferation and CD40L expression (139). miR-153-3p also targets Peli1 to reduce Tfh cell accumulation in patients with systemic lupus erythematosus (140). Meanwhile, Taganov et al. demonstrated that miR-146a expression is up-regulated by the NF-κB pathway and itself directly targets TRAF6 and IRAK1 to create a negative feedback loop to prevent the overactivation of Tfh cell (141).

Other miRNAs and their target genes are involved in Tfh cell function (**Figure 5**). For example, miR-92a promotes Tfh precursor induction in T1D islet autoimmunity by directly targeting Krüppel-like factor 2 (KLF2), thus suppressing PTEN-PI3K-KLF2 signaling, increasing immune infiltration and activation in pancreas (142). miR-663 upregulates Tfh cell and downregulates Treg cell by targeting TGF-β1, which induces immune dysregulation in patients with systemic lupus erythematosus (143). Wang et al. demonstrated that miR-7 not only downregulates PTEN/AKT signaling, which promotes B cell differentiation into plasmablasts/plasma cells and spontaneous germinal center formation, but also increases phosphorylated STAT3 and IL-21 expression to facilitate Tfh expansion and promote systemic lupus erythematosus development (144). miR-346 directly targets Bcl-6 to promote Tfh cell differentiation in the pathogenesis of Graves’ disease, whereas Bcl-6 binds to the miR-31 promoter to repress its transcription, leading to the release of CD40L and SAP, which contribute to Tfh function. These results identify a miRNA-Bcl-6 positive feedback loop that stabilizes the Tfh cell program (145, 146). However, genome-wide miRNA expression profiling showed that miR-126-3p represses Tfh cell retention in germinal center by inhibiting S1PR2 expression, which is curial for angioimmunoblastic T-cell lymphoma pathobiology (147). miR-15b (148) and miR-192 (149) also affect Tfh cell differentiation and accumulation by directly targeting IRF4 and CXCR5, respectively. Other miRNAs related to Tfh cell differentiation and fate decision are shown in **Supplementary Table 5**.

### 3.5 miRNAs in Treg cell differentiation and fate decision

Treg cells play irreplaceable roles in controlling immune responses by suppressing the function of “good immune cells,” such as Th cells, cytotoxic T lymphocytes, B cells, natural killer cells, and antigen-presenting cells, thus serving as “brakes” to maintain immune homeostasis (57). The characteristic feature of Treg cells is the expression of the master transcription factor Foxp3. Nowadays, two kinds of Foxp3^+^ Treg cells have been revealed, the natural CD4^+^CD25^+^ Treg (nTreg) cells raised in the thymus and the TGF-β induced Treg (iTreg) cells generated in the periphery (150). In general, Treg cells are iTreg cells. The activation of IL-2/STAT5 and TGF-β/SMAD2/3 signaling lead to the expression of Foxp3. However, the IL-6/STAT3 signaling pathway negatively regulates Foxp3 expression via STAT3. In addition to p-SMAD2/3 and p-STAT5, the transcription of Foxp3 is regulated by other transcription factors, such as HIF-1α and AP1. Moreover, Foxp3 cooperates with Foxo1 and NFAT to induce CTLA4 transcription and expression, thereby suppressing the function of the key co-stimulation factor CD28 (2).

**Figure 6:**
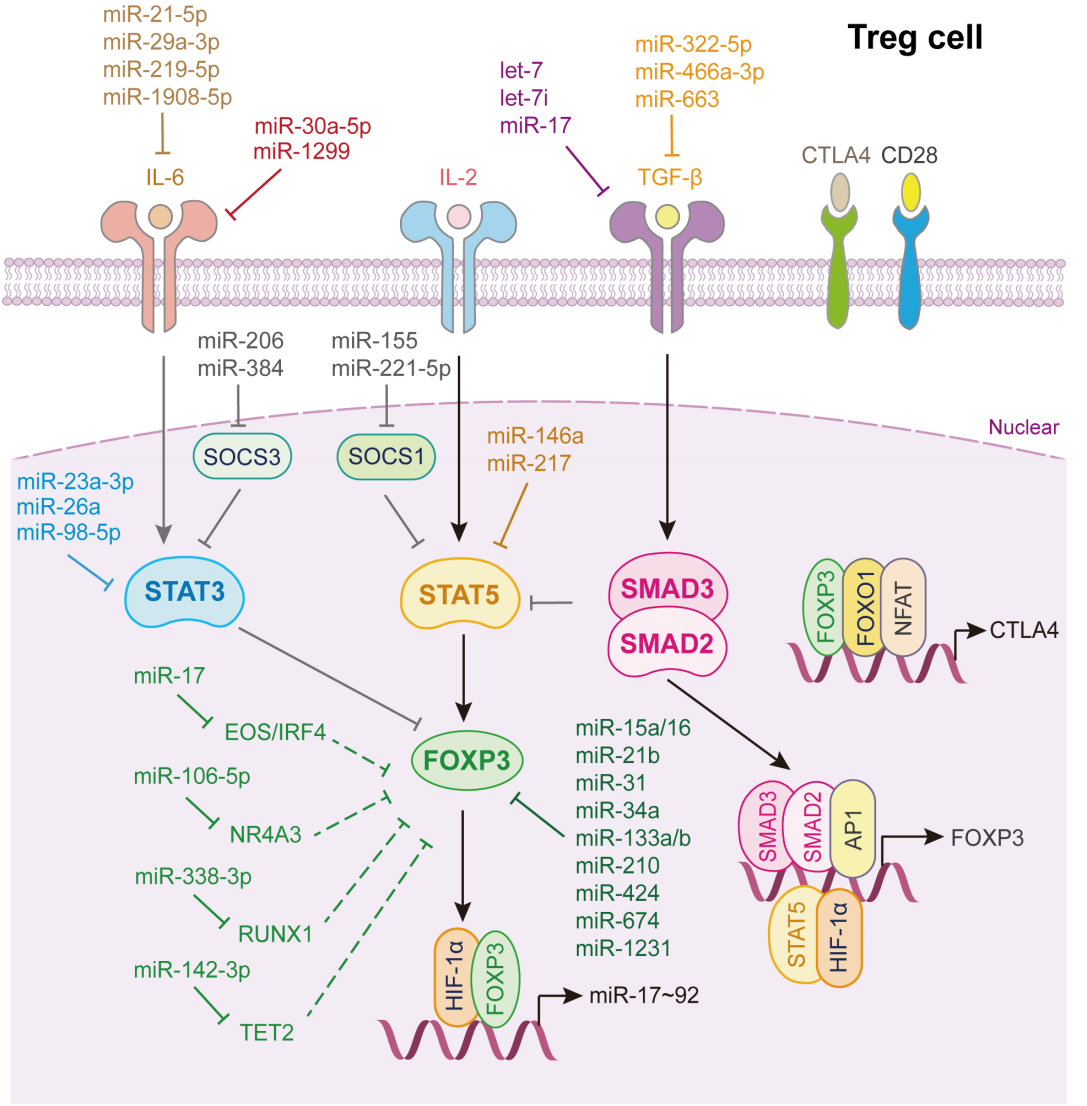
miRNAs in Treg cell differentiation and fate decision. IL-6 or IL-2, interleukin-6 or 2; TGF-β, transforming growth factor beta 1; STAT3 or STAT5, signal transducer and activator of transcription 3 or 5; FOXP3, Forkhead box P3; SMAD2 or SMAD3, SMAD Family Member 2 or 3; HIF-1α, hypoxia inducible factor 1 subunit alpha; AP1, Jun proto-oncogene; NFAT, nuclear factor of activated T-cells; FOXO1, Forkhead box O1; CTLA4, cytotoxic T-lymphocyte associated protein 4.

As shown in **Figure 6**, many miRNAs regulate Treg cells differentiation. Notably, miR-15a/16 (151), miR-31 (152), miR-34a (119), miR-21b (153), miR-133a/b (154), miR-210 (155), miR-424 (156), miR-674, and miR-1231 (152) directly bind to the 3’UTR of Foxp3 mRNA and inhibit its expression. Among them, miR-15a/16 impairs umbilical cord blood-derived Treg, which alleviates the progression of graft-versus-host disease (151), while, miR-21b disturbs the Th17/Treg balance to inhibit the progression of acute graft-versus-host disease after allogeneic hematopoietic stem cell transplantation (153). Rouas et al. revealed that miR-31 is considerably under-expressed in human natural Treg cell, and negatively regulates the differentiation and activation of Treg cell (152). Zhao et al. showed that miR-210 level is increased in CD4^+^ T cells from patients with psoriasis vulgaris, which induces immune dysfunction and contributes to disease progression (155).

As for TGF-β signaling, let-7i (157), let-7 (158), miR-17 (32), miR-663 (143), miR-322-5p (129) and miR-466a-3p (159) directly target either TGF-β or its receptors to restrain TGF-β signaling activation which strongly restricts Treg cells activation. Through whittling TGF-β signaling and Treg cell induction, let-7i, up-regulated in CD4^+^ T cells from patients with multiple sclerosis, modulates the pathogenesis of multiple sclerosis (157), while loss of Lin28b expression in fetal T cells leads to increased mature let-7, which reduces fetal T cells differentiate into Treg cells (158). Geng et al. illustrated that patients with systemic lupus erythematosus express a specific miRNA, miR-663, in bone marrow-derived mesenchymal stem cells (BMSCs). It inhibits the proliferation and migration of BMSCs and the activation of BMSCs derived Treg cell, which induces immune dysfunction in systemic lupus erythematosus (143) (**Figure 6**).

Additionally, miR-23a-3p (81), miR-26a (82), miR-98-5p (83) directly target STAT3, whereas miR-29a-3p (94), miR-21-5p (94), miR-219-5p (160) and miR-1908-5p (99) target IL-6 to inhibit Treg cells differentiation. miR-1299 and miR-30a-5p reduce their transcript target levels, IL-6R and IL6ST, and subsequently increase Treg accumulation (84). miR-146a (161) and miR-217 (162) bind directly to STAT5 and repress its expression, thereby inhibiting Treg cells activation (**Figure 6**).

miRNAs also control the expression of other factors, influencing Treg cells differentiation (**Figure 6**). miR-338-3p, miR-106-5p, miR-142-3p, and miR-17 target RUNX1 (163), nuclear receptor subfamily 4 group A member 3 (164), Tet2 (165), and Eos/IRF4 (166), respectively, to further alleviate Foxp3 expression, thus restricting Treg cell fate decision. miR-15b/16 (167) and miR-342-3p (168) enhance the induction of Treg cells by regulating the expression of Rictor, also known as mammalian target of rapamycin complex 2, which is required for AKT activation. miR-99a and miR-150 hamper Th17 and Treg activation by reducing mTOR expression (34). Interestingly, Treg cells can provide feedback to control their activation by secreting several miRNAs. Xie et al. showed that miR-34a was downregulated in iTreg cells but upregulated in Th17 cells, disrupting the Treg/Th17 balance. Mechanistically, IL-6 or TNF-α activates p65, which binds to the miR-34a promoter. The upregulation of miR-34a transcription suppresses Foxp3 expression (119).

Additionally, Treg cell fate decision is regulated by miRNAs from other immune cells and cancer cells (**Figure 6**). Ning et al. found that miR-208b in the exosomes of colon cancer cells promotes Treg expansion by targeting programmed cell death factor 4, thus resulting in tumor growth *in vivo (* 169). Malignant pleural effusion is a special metastatic tumor entity in patients with cancer. Single-cell sequencing data showed that exosomes released from macrophages in malignant pleural effusion can promote the differentiation of naïve T cells into Treg cells by upregulating miR-4443, which influences protein kinase B and lipid biosynthetic processes (170). Other miRNAs involved in regulating Treg cell differentiation and fate decision are displayed in **Supplementary Table 6**.

## 4 miRNAs in the Teff/Treg balance

Because many master transcription factors and related intracellular signal transduction cascades overlap among different Th cell subtypes, the balance between Teff and Treg cells largely orchestrates the outcome of immune responses against pathogens or tumor cells.

A typical example is the Th17/Treg balance, as IL-6/STAT3 signaling activates Th17 cells while IL-2/STAT5 inhibits them, the activation pathways in Treg cells are opposite to those in Th17 cells, except for TGF-β/SMAD2/3 signaling which promotes both Th17 and Treg cells activation (**Figures 4**, **6**). Therefore, most miRNAs regulate IL-6/STAT3 and IL-2/STAT5 signaling in both Th17 and Treg cells, affecting the Th17/Treg cells balance. For example, miRNAs directly targeting IL-6, IL-6R, or STAT3 contribute to Treg cells, while restraining Th17 cells differentiation, which decreases the Th17/Treg balance. In contrast, miRNAs targeting IL-2 or STAT5 have the opposite effect on the Th17/Treg balance (**Figures 4**, **6**).

miRNAs regulate the expression of other transcription factors, thereby affecting the Th17/Treg balance (**Figure 6**). SOCS1, which is a negative regulator of STAT5 activation, is a target of miR-155 (171) and miR-221-5p (103). SOCS3, which is a negative regulator of STAT3 activation, is a target of miR-384 (107) and miR-206 (104). Anusara et al. found that Sirtuin-1-deficient T cells promote iTreg cells differentiation and inhibit IFN-γ production, indicating Sirt-1 facilitates the Th17/Treg balance, which contributes to the induction of acute graft-versus-host disease (172). Further studies showed that miR-23a-3p (173) and miR-155 (174) disturbed the Th17/Treg balance by targeting Sirt1, which modulate the progression of Graves’ disease and chronic periodontitis, repectively. Chen et al. demonstrated that miR-485-5p inhibition suppressed Treg cells and the Treg/Th17 balance, but elevated Th17 cells, by targeting Absent in melanoma 2 (175).

Additionally, the Treg/Th17 balance is regulated by miRNAs from other immune cells and cancer cells. Exosomal miR-23b-3p derived from human bone marrow mesenchymal stem cells inhibits the activation of the PI3K/Akt/NF-κB signaling pathway. It reduces the number of Th17 cells to maintain the balance of Th17/Treg cells in intracranial aneurysms by targeting KLF5 (176). Ji et al. also indicated that exosomes derived from thymic stromal lymphopoietin-treated dendritic cells enhance RORγt and IL-17 while repressing Foxp3 and IL-10 expression in CD4^+^ T cells. Further studies have shown that miR-21 is highly expressed in exosomes and suppresses Smad7 expression, thereby disrupting Treg/Th17 differentiation (177).

Although the Treg/Th17 balance is the most studied aspect of the Treg/Teff balance, the Treg/Th1, Treg/Th2, and Treg/Tfh balance can also can be regulated by several miRNAs. For example, Kelada et al. used an in-silico analysis to identify miR-10a and miR-182 as critical miRNAs in Th1- and Th2-associated Treg cells during Schistosome and Leishmania-associated inflammation. Further investigation indicated that the IL-12/IFN-γ axis reduces miR-10a and its target gene CREB in Th1-associated Treg cells, promoting their function. In contrast, IL-4 regulates miR-182 and c-Maf in Th2-associed Treg cells, which attenuates Th2 function but augments Treg function (178). Another classical example is miR-17-92, which regulates the Th1/Treg balance. Th1 cell response is promoted whereas Treg cell differentiation is restricted by miR-19b/PTEN and miR-17/TGFβRII/CREB1, which alleviates tumor evasion (32). Geng et al. revealed that patients with systemic lupus erythematosus display a unique miRNA, miR-663, in their bone marrow stromal cells. Mechanically, miR-663 impairs the bone marrow stromal cells-mediated downregulation of Tfh cells and upregulation of Treg cells by targeting TGF-β1, thus affecting the Tfh/Treg balance and aggravating lupus (143).

## 5 miRNAs in Th cell-mediated tumor immunity

Tumors develop multiple potent and overlapping mechanisms to mediate immune evasion, one of which is increasing Treg cell accumulation or disturbing the Teff/Treg balance in the TME or TIME (179). Previous studies have shown that many miRNAs are involved in the regulation of Treg cells and the Teff/Treg balance; however, only a small number of miRNAs have been shown to play roles in Th cell-mediated tumor immunity.

First, some miRNAs influence Th17-mediated tumor immunity. miR-130b directly targets the IFNAR1/p-STAT1 pathway to recruit Th17 cell and promotes its tumor-suppressive role via the OX40/OX40L interaction in diffuse large B-cell lymphoma. This regulation alters the TME and facilitates tumor progression (180). Zhou et al. found that tumor-associated macrophages secrete miRNAs such as miR-29-3p and miR-21-5p to increase the Treg/Th17 imbalance, which generates an immune-suppressive microenvironment and facilitates epithelial ovarian cancer progression (94). Interestingly, miR-21 also contributes to the immuno-suppressive microenvironment in multiple myeloma (181). Rossi et al. indicated that the inhibition of miR-21 in naïve T cells impaired Th17 cell differentiation, upregulated STAT1/STAT5a/5b expression, and redirected Th17 to Th1/Th2-like activated/polarized cells, thereby abrogating Th17-mediated multiple myeloma cell proliferation and osteoclast activity. miR-98-5p and miR-451 reshaped the TME in gastric cancer by influencing Th17 cell distribution and differentiation (83, 182). In addition, miR-146a attenuates Th17 cell differentiation to promote cervical cancer cell growth and represses its apoptosis through NF−κB signaling by targeting TRAF6 (125).

Second, other miRNAs may affect Treg cell-mediated tumor immunity. Yin et al. revealed that miR-214 expression is elevated in breast cancer, hepatocellular carcinoma, non-small-cell lung cancer, and pancreatic cancer. Further studies indicated that tumor-derived miR-214 efficiently downregulates PTEN and promotes Treg cell expansion, thereby enhancing immune suppression and tumor implantation/growth in mice (179). Wei et al. reported that miR-124 reverses the glioma stem cell-mediated immunosuppression of T cell proliferation and Treg induction to enhance T cell-mediated immune clearance by inhibiting the STAT3 pathway (183). Ye et al. showed the exosomal miR-24-3p is involved in tumor pathogenesis; it mediates T-cell suppression via the repression of FGF11 and may serve as a potential prognostic biomarker in nasopharyngeal carcinoma (184). In addition, miR-448 (185), miR-302a (186), miR-208b (169), miR-192-5p (187), miR-497 (188), and miR-325-3p (189) affect tumor immunity by effectively regulating Treg cell activation or the Treg/Teff cell balance in breast cancer, glioma, colon cancer, gastric cancer, colorectal cancer, and oral squamous cancer.

## 6 Discussion

Since first discovered in 1993, miRNAs have been shown to participate in almost all biological processes, including Th cell-mediated immune response, through RNA-sequencing (2, 3, 8, 190). In this review, we comprehensively summarize the roles of miRNAs in Th cell development, activation, differentiation, and fate decisions, as well as in tumor immunity (**Figures 1**–**6**; **Supplementary Tables 1**-**6**). Previous studies have indicated that master transcription factors are the main players in Th cell function (1); however, our review reveals that miRNAs, which target numerous target genes, are also flexible and accurate regulators of Th cell fate decisions and the Teff/Treg balance. This review broadens our understanding of the role of miRNAs in Th cell development, differentiation, activation, fate decisions and tumor immunity. It also emphasizes that miRNAs play vital roles in regulating immune responses against pathogens and cancer cells.

Analyzing of the miRNAs involved in Th cell function revealed some interesting aspects. First, we found that one miRNA can regulate more than one subset of Th cell functions. The miR-17-92 cluster not only controls T cell development in the long-term HSC self-renewal, DN2a to DN2b, and DN4 to DP steps, but also affects all subsets of Th cells by targeting PTEN. This triggers the activation of PI3K/AKT/mTOR signaling (134, 135, 191–193), which is essential for cell survival and co-stimulation during Th cell differentiation and activation. Another example is miR-155, which participates in all Th subset cells differentiation by regulating various genes. It directly targets IFN-γ receptor alpha thus contributing to Th1 cell differentiation (47). Conditional knockout mice showed that miR-155 in T cells suppressed c-Maf, SOCS1, Fosl2 and Jarid2 expression in Th2 cell, while suppressing C/EBPβ in Th17 cell, attenuating Th2 and Th17 cells activation (67). In Tfh cell, miR-155 represses Peli1 expression and increases cellular proliferation and CD40 ligand expression, thus facilitating Tfh cell differentiation (139). miR-155 enhances Treg cell but inhibits Th17 cell by targeting SIRT1 in chronic periodontitis (174).

Second, many different miRNAs collectively control gene expression during Th cell function. Foxp3, a master transcription factor involved in Treg cell differentiation and activation, is regulated by more than 14 miRNAs (**Figure 6**; **Supplementary Table 6**). Among them, ten miRNAs (miR-15a/16 (151), miR-31 (152), miR-34a (119), miR-21b (153), miR-133a/b (154), miR-210 (155), miR-424 (156), miR-674 (152), and miR-1231 (152)) directly targeted Foxp3, while four miRNAs (miR-338-3p (163), miR-106-5p (164), miR-142-3p (165), and miR-17 (166)) targeted other genes to affect Foxp3 expression. A similar phenomenon was observed in Th17 cell activation. Nine miRNAs (let-7f-5p (93), miR-29a-3p (94), miR-21-5p (94), miR-93-5p (54), miR-20b (95), miR-199a-5p (96), miR-125a/b (97), miR-302c-5p (98) and miR-1908-5p (99)) bind to the 3’UTR of STAT3 to inhibit its expression, which represses Th17 cell differentiation and activation (**Figure 4**).

Third, although most miRNAs are conserved between humans and mice, some have opposing roles in human and mouse Th cells. For example, miR-21 (181) and miR-21-5p (194) promote Th17 activation in mice, but inhibit Th17 activation in humans (177, 195) (**Supplementary Table 4**). As extensive studies on miRNAs function in Th cells have been conducted in mouse models, more efforts should be made to verify their roles in humans. These findings indicate that miRNAs cooperate to concisely and duly control Th cell differentiation and activation depending on the environment.

Four, since miRNAs play vital roles in the development, differentiation, activation and fate decision of Th cells, the dysregulation of these miRNAs facilitates the progression of many inflammatory diseases. Therefore, using antagomirs or mimics of these miRNAs will be a good strategy to increase the therapy responses. For example, when MRL^lpr^/^lpr^ lupus mice are administrated with miR-7 antagomir or miR-663 inhibitor, the manifestations of systemic lupus erythematosus in mice are efficiently improved by regulating Tfh/Treg imbalance (143, 144). He et al. reported that application of antisense miR-301a in mouse colitis model significantly decreases the numbers of Th17 cell and amounts of pro-inflammatory cytokines in inflamed colon, thus releasing the manifestations of inflammatory bowel disease (128). Wu et al. illustrated that both the ablation of miR-210 in mice and inhibition of miR-210 by intradermal injection of antagomir-210 block the immune imbalance and the development of psoriasis-like inflammation in an imiquimod-induced or IL-23-induced psoriasis-like mouse model. Given that the treatment effects of these miRNAs are partly validated *in vivo*, so more efforts should be taken to clarify the potential roles of these miRNAs on the treatment of inflammatory diseases.

Finally, we noticed that miRNAs loaded in exosomes derived from other immune cells or cancer cells affect the expression of genes involved in Th cell activation, which leads to the dysfunction of Teff/Treg balance and changes the TME or TIME. In “cold” tumors, Teff cells fail to infiltrate the tumor and activate immune responses. Therefore, exosome-loaded miRNAs that contribute to Teff cells and/or repress Treg cells may be good candidates for future cancer treatment. One promising example is the delivery of anti-miR-214 micro-vesicles into mice implanted with tumors that block Treg expansion and inhibit tumor growth derived from lung cancer and sarcoma (179). Additionally, Ning et al. demonstrated that colon cancer cell-secreted miR-208b is sufficiently delivered into recipient T cells to promote Treg expansion, thus resulting in the tumor growth and oxaliplatin resistance in colon cancer (169). Therefore, targeting miRNAs therapy may be a good approach for immunotherapy.

In a word, although approximately 300 miRNAs have been shown to control Th cell function, only a limited number of miRNAs are verified to regulate Th cell-mediated tumor immunity or inflammatory disease *in vivo*. We believe that studies on the role of miRNAs in inflammatory disease, in the TIME, and in cancer treatment are ongoing. The remaining questions include which miRNAs should be included and what combination of these miRNAs achieve the best efficacy in the treatment of inflammatory disease and cancer, which warrants further investigation.

## Author contributions

S-JX: Conceptualization, Funding acquisition, Investigation, Writing – original draft. J-HC: Validation, Writing – review & editing. SC: Funding acquisition, Supervision, Validation, Writing – review & editing. H-LL: Supervision, Validation, Writing – review & editing.
