# Supplementary material for: The role of miRNAs in T helper cell development, activation, fate decisions and tumor immunity
Source: Front Immunol. 2024 Jan 9;14:1320305. doi: 10.3389/fimmu.2023.1320305 (PMC10803515; doi:10.3389/fimmu.2023.1320305)
Supplement: Supplementary file 1 [file Table_1.docx]

**Supplementary Table 1.** miRNAs in T cell development.

| **Human** | | **Mouse** | |
| --- | --- | --- | --- |
| miR-17 (1) (+) | miR-99(2) (-) | miR-126(3)(+) | miR-126a (4)(-) |
| miR-125b(5)(+) | miR-29a(6) (-) | miR-125a (7) (+) | miR-21 (8)(-) |
| miR-22 (9) (+) | miR-221/222 (10) (-) | miR-125b(11)（+） | miR-99(2) (-) |
| miR-143/145 (12) (+) | miR-10a/335 (13) (-) | miR-22 (9) (+) | miR-29a(6) (-) |
| miR-9(14)(+) | miR-150 (15) (-) | miR-143/145 (12) (+) | miR-193b(16) (-) |
|  | miR-185 (17) (-) | miR-146a (18) (+) | miR-23a/23b (19) (-) |
|  | miR-181c(20)(-) | miR-181a (21) (+) | miR-127-3p (22) (-) |
|  |  | miR-205 (23) (+) | miR-146b (24) (-) |
|  |  | miR-223 (25) (+) | miR-196b (26, 27) (-) |
|  |  | miR-142-3p (28) (+) | miR-17-92 (29) (30) (-) |
|  |  | Dicer (31) (+) | miR-150 (32) (-) |
|  |  | Dicer-1 (33) (+) | miR-23a (34) (-) |
|  |  |  | miR-181d (35) (-) |
|  |  |  | miR-185 (17) (-) |
|  |  |  | mmu-miR-10 (27) (-) |

Note: (+): promote the process; (–): suppress the process.

**Supplementary Table 2.** miRNAs in Th1 cell differentiation, activation and fate decision.

| **Human** | | **Mouse** | |
| --- | --- | --- | --- |
| miR-210(36)(+) | miR-219a-5p(37)(-) | miR-155 (38-41) (+) | miR-10a (42, 43) (-) |
| miR-182(44)(+) | miR-140-5p(45) (-) | miR-142(46)(+) | miR-29(47)(-) |
| miR-92a (48)(+) | miR-500a-5p(49)(-) | miR-4281 (50) (+) | miR-21(51)(-) |
| miR-125b(52)(+) |  | miR-17-92 (53-55) (+) | miR-140-5p(45) (-) |
| miR-26a/b(56)(+) |  | miR-181b-5p/30b*(57) (+) | miR-146a(58)(-) |
| miR-494(59)(+) |  | miR-210(36)(+) | Dicer(33)(-) |
|  |  | miR-182(60)(+) | Drosha(61)(-) |
|  |  | miR-92a (48)(+) | miR-93-5p(62)(-) |
|  |  | miR-125a(63, 64)(+) | miR-29a-3p(62) (-) |
|  |  | miR-24(65)(+) | let-7d(66)(-) |
|  |  | miR-17(53)(+) | miR-122a-5p(67) (-) |
|  |  | miR-148a(68)(+) | miR-99a(69)(-) |
|  |  | miR-126a-5p(70)(+) | miR-19a(71)(-) |
|  |  | miR-374b-5p (72)(+) |  |
|  |  | miR-23a(73)(+) |  |
|  |  | miR-146a-5p(74)(+) |  |
|  |  | miR-345-5p(75)(+) |  |
|  |  | miR-381-3p(76)(+) |  |
|  |  | miR-124(77)(+) |  |

Note: (+): promote the process; (–): suppress the process.

**Supplementary Table 3**. miRNAs in Th2 cell differentiation, activation and fate decision.

| **Human** | **Mouse** | |
| --- | --- | --- |
| miR-210(36)(-) | miR-21(78)(+) | miR-210(36)(-) |
| miR-146a-5p(79)(-) | miR-155(80)(+) | miR-122a-5p (67)(-) |
| miR-135b(81)(-) | miR-27b(82)(+) | miR-345-5p(75)(-) |
| miR-29c(83)(-) | miR-206(82)(+) | miR-340(84)(-) |
| miR-29b(85)(-) | miR-106b(82)(+) | miR-29b(85)(-) |
| miR-451(86)(-) | miR-203(82)(+) | miR-466a-3p(87)(-) |
| miR-135b-5p(88)(-) | miR-23b(82)(+) | miR-495(89)(-) |
| miR-124-3p(90)(-) | miR-19a(71)(+) | miR-124(77, 90)(-) |
|  | miR-21(91)(+) | miR-19b(92)(-) |
|  | miR-17-92(55)(+) | miR-24/27(93)(-) |

Note: (+): promote the process; (–): suppress the process.

**Supplementary Table 4.** miRNAs in Th17 cell differentiation, activation and fate decision.

| **Human** | | **Mouse** | |
| --- | --- | --- | --- |
| miR-873 (94) (+) | miR-21-5p(95) (-) | miR-92a(48)(+) | miR-467b (96)(-) |
| miR-425(97)(+) | miR-21(98)(-) | miR-425(97)(+) | miR-126(99)(-) |
| miR-210(36)(+) | miR-1908-5p(100)(-) | miR-184(101)(+) | miR-93-5p(62)(-) |
| miR-34a(102)(+) | let-7f(103, 104)(-) | miR-183(105)(+) | miR-29a-3p(62)(-) |
| miR-182(44) (+) | let-7a(106)(-) | miR-96(105)(+) | miR-125a/b(107)(-) |
| miR-381-3p(76)(+) | miR-29(108)(-) | miR-182(105)(+) | miR-199a-5p(109)(-) |
| miR-206(110)(+) | miR-17(111)(-) | miR-210(36)(+) | miR-302c-5p(112)(-) |
| miR-130b(113)(+) | miR-29c(83)(-) | miR-21(114)(+) | miR-20b(115)(-) |
| miR-451(116)(+) | miR-181a(117)(-) | miR-21-5p(118)(+) | let-7g-5p(119)(-) |
| miR-149-3p(120)(+) | miR-23a-3p(121)(-) | miR-34a(102)(+) | miR-34a(122)(-) |
| miR-155(123)(+) | miR-23b-3p(124)(-) | miR-223-3p(125)(+) | miR-17-5p(126)(-) |
| miR-802(127)(+) | miR-219a-5p(37)(-) | miR-490(128)(+) | miR-17-92(129)(-) |
| miR-301a(130)(+) | miR-10a-3p(131)(-) | miR-182(60) (+) | miR-124-3p(132)(-) |
| miR-101-3p(133)(+) | miR-10a(43)(-) | miR-374b-5p(72)(+) | miR-23b(134)(-) |
| miR-19b-3p(135)(+) | miR-29b(108)(-) | miR-23a(73)(+) | miR-340(136)(-) |
| miR-31-5p(137)(+) | miR-30a-5p(138, 139)(-) | miR-10a(140)(+) | miR-3082-5p(141)(-) |
| miR-1-3p(142)(+) | miR-1246(143)(-) | miR-24(65)(+) | miR-1192(128)(-) |
| miR-542-5p(144)(+) | miR-29a-3p(145)(-) | miR-212/132(146)(+) | miR-466i(147)(-) |
| miR-217(148)(+) | miR-326(149)(-) | miR-155(150)(+) | miR-30a-5p(138)(-) |
| miR-141-3p(151)(+) | miR-125a(152)(-) | miR-146/155(153)(+) | miR-1299(138)(-) |
| miR-200a-3p(151)(+) | miR-125b(154)(-) | miR-448(155)(+) | miR-124(156)(-) |
| miR-218-5p(157)(+) | miR-374b-5p(158)(-) | miR-326(159)(+) | miR-3082-5p(141)(-) |
| miR-217(148)(+) | miR-106-5p(158)(-) | miR-181c(160)(+) | miR-181a(117)(-) |
| miR-141-3p(151)(+) | miR-302c-5p(112)(-) | miR-31(161)(+) | miR-26a(162)(-) |
| miR-200a-3p(151)(+) | miR-378g(163)(-) | miR-384(164, 165)(+) | miR-221-5p(166)(-) |
| miR-218-5p(157)(+) | miR-18a(167)(-) | miR-409-3p(168)(+) | miR-221/222(169)(-) |
| miR-146a-5p(170)(+) | miR-181a(171)(-) | miR-1896(168)(+) | miR-221(172)(-) |
| miR-106b-5p(173)(+) | miR-98-5p(174)(-) | miR-322-5p(175)(+) | miR-302c-5p(112)(-) |
|  | miR-181a(171)(-) | miR-686(176)(+) | miR-30a(139, 177)(-) |
|  | miR-98-5p(174)(-) | miR-424(178)(+) | miR-301a(179)(-) |
|  | miR-183-5p(180)(-) |  | miR-20a(181)(-) |
|  | miR-99a(180)(-) |  | miR-18b(182)(-) |
|  | miR-146a(183)(-) |  | miR-106a(182)(-) |
|  | miR-146a-5p(184)(-) |  | miR-363-3p(182)(-) |
|  |  |  | miR-449a-5p(185)(-) |
|  |  |  | miR-182-5p(186)(-) |
|  |  |  | miR-26-5p(187)(-) |
|  |  |  | miR-485(188)(-) |
|  |  |  | miR-18a(167)(-) |
|  |  |  | miR-132(189)(-) |
|  |  |  | miR-183(190)(-) |
|  |  |  | miR-590-3p(191)(-) |
|  |  |  | miR-15b(192)(-) |
|  |  |  | miR-374c(193)(-) |
|  |  |  | miR-365-3p(194)(-) |

Note: (+): promote the process; (–): suppress the process.

**Supplementary Table 5**. miRNAs in Tfh cell differentiation, activation and fate decision.

| **Human** | | **Mouse** | |
| --- | --- | --- | --- |
| miR-92a(195)(+) | miR-146a(196)(-) | miR-17-92(55, 197)(+) | miR-146a/b(198)(-) |
| miR-18a/17(199)(+) | miR-126-3p(200)(-) | miR-155(41, 201)(+) | miR-15b(202)(-) |
| miR-153-3p(203)(+) | miR-29a-3p(204)(-) | miR-21(205)(+) | miR-23-27-24 cluster(206)(-) |
|  | miR-192(207)(-) | miR-663(208)(+) |  |
|  | miR-31-5p(209)(-) | miR-7(210)(+) |  |
|  | miR-346(211)(-) |  |  |

Note: (+): promote the process; (–): suppress the process.

**Supplementary Table 6.** miRNAs in Treg cell differentiation, activation and fate decision.

| **Human** | | **Mouse** | |
| --- | --- | --- | --- |
| miR-29a-3p/21-5p(145)(+) | miR-34a(102)(-) | miR-219-5p(212)(+) | miR-34a(102)(-) |
| miR-1908-5p(100)(+) | miR-21b(213)(-) | miR-221-5p(166)(+) | miR-424(178)(-) |
| miR-146a-5p(184, 214)(+) | miR-133a/b(215)(-) | miR-155(216)(+) | miR-210(217)(-) |
| miR-146a(218) (+) | miR-325-3p(219)(-) | miR-19b/17(53)(+) | miR-15a/16(220)(-) |
| miR-19b(221)(+) | miR-31(222)(-) | miR-214(223)(+) | miR-17(224)(-) |
| miR-98-5p(174)(+) | miR-210(225)(-) | miR-26a(162)(+) | miR-384(165)(-) |
| miR-23a-3p(121)(+) | miR-31/674/1231(226)(-) | miR-181a/b-1(227)(+) | miR-146a(228)(+) |
| miR-155-5p(229)(+) | miR-338-3p(230)(-) | miR-181a-5p(231)(+) | miR-155(232)(-) |
| miR-33a/33b/181a(233)(+) | miR-106b-5p(173)(-) | miR-132(189)(+) | miR-181a(234)(-) |
| miR-23a-3p(235)(+) | miR-142-3p(236)(-) | miR-10a(42)(+) | miR-20a(237)(-) |
| miR-485-5p(238)(+) | miR-206(110)(-) | miR-342-3p(239)(+) | miR-99a/150(240)(-) |
| miR-17(111)(+) | miR-146a/155(241)(-) | miR-15b/16(242)(+) | miR-322-5p(175)(-) |
| miR-31(243)(+) | let-7i(244)(-) | miR-682(245)(+) | miR-466a-3p(246)(-) |
| miR-10a-3p(131)(+) | let-7(247)(-) | miR-302(248)(+) | miR-31(249, 250)(-) |
| miR-448(251)(+) | let-7f(103)(+) | miR-142(252)(+) | miR-10a/182(253)(-) |
| miR-192-5p(254)(+) | miR-663(208)(-) | miR-15a/16-1(255)(+) | miR-23b-3p(256)(-) |
| miR-378g(163)(+) | miR-217(148)(-) | miR-17-92(257)(+) | miR-92a(48)(-) |
| miR-125b(154)(+) | miR-99a(180)(-) | miR-224(258)(+) |  |
| miR-208b(259)(+) | miR-568(260)(-) | miR-126a(261)(+) |  |
| miR-125a-5p(262)(+) | miR-23b-3p(124)(-) | Dgcr8(263)(+) |  |
| miR-4443(264)(+) | miR-103(265)(-) | pri-miRNA-31(266)(+) |  |
| miR-193-3p(267)(+) | miR-21(98)(-) |  |  |
|  | miR-363(268)(-) |  |  |
|  | miR-124(269)(-) |  |  |
|  | miR-19b-3p(135)(-) |  |  |
|  | miR-302a(270)(-) |  |  |
|  | miR-92a(48)(-) |  |  |
|  | miR-497(271)(-) |  |  |
|  | miR-4772-3p(272)(-) |  |  |
|  | miR-1246(143)(-) |  |  |
|  | miR-153-3p(203)(-) |  |  |
|  | miR-24-3p(273)(-) |  |  |

Note: (+): promote the process; (–): suppress the process.

1. Yang Y, Ma W, Wu D, Huang Y, Li H, Zou J, et al. MiR-17 partly promotes hematopoietic cell expansion through augmenting HIF-1alpha in osteoblasts. PLoS One. 2013;8(7):e70232. doi:10.1371/journal.pone.0070232

2. Khalaj M, Woolthuis CM, Hu W, Durham BH, Chu SH, Qamar S, et al. miR-99 regulates normal and malignant hematopoietic stem cell self-renewal. J Exp Med. 2017;214(8):2453-70. doi:10.1084/jem.20161595

3. Hu L, Xu H, Lu J, Zhou Y, Chu F, Zheng W, et al. MicroRNA-126 Deficiency Affects the Development of Thymus CD4+ Single-Positive Cells through Elevating IRS-1. Int Arch Allergy Immunol. 2018;177(3):207-18. doi:10.1159/000490710

4. Lechman ER, Gentner B, van Galen P, Giustacchini A, Saini M, Boccalatte FE, et al. Attenuation of miR-126 activity expands HSC in vivo without exhaustion. Cell Stem Cell. 2012;11(6):799-811. doi:10.1016/j.stem.2012.09.001

5. Ooi AG, Sahoo D, Adorno M, Wang Y, Weissman IL, Park CY. MicroRNA-125b expands hematopoietic stem cells and enriches for the lymphoid-balanced and lymphoid-biased subsets. Proc Natl Acad Sci U S A. 2010;107(50):21505-10. doi:10.1073/pnas.1016218107

6. Hu W, Dooley J, Chung SS, Chandramohan D, Cimmino L, Mukherjee S, et al. miR-29a maintains mouse hematopoietic stem cell self-renewal by regulating Dnmt3a. Blood. 2015;125(14):2206-16. doi:10.1182/blood-2014-06-585273

7. Wojtowicz EE, Broekhuis MJC, Weersing E, Dinitzen A, Verovskaya E, Ausema A, et al. MiR-125a enhances self-renewal, lifespan, and migration of murine hematopoietic stem and progenitor cell clones. Sci Rep. 2019;9(1):4785. doi:10.1038/s41598-019-38503-z

8. Hu M, Lu Y, Zeng H, Zhang Z, Chen S, Qi Y, et al. MicroRNA-21 maintains hematopoietic stem cell homeostasis through sustaining the NF-kappaB signaling pathway in mice. Haematologica. 2021;106(2):412-23. doi:10.3324/haematol.2019.236927

9. Song SJ, Ito K, Ala U, Kats L, Webster K, Sun SM, et al. The oncogenic microRNA miR-22 targets the TET2 tumor suppressor to promote hematopoietic stem cell self-renewal and transformation. Cell Stem Cell. 2013;13(1):87-101. doi:10.1016/j.stem.2013.06.003

10. Felli N, Fontana L, Pelosi E, Botta R, Bonci D, Facchiano F, et al. MicroRNAs 221 and 222 inhibit normal erythropoiesis and erythroleukemic cell growth via kit receptor down-modulation. Proc Natl Acad Sci U S A. 2005;102(50):18081-6. doi:10.1073/pnas.0506216102

11. O'Connell RM, Chaudhuri AA, Rao DS, Gibson WS, Balazs AB, Baltimore D. MicroRNAs enriched in hematopoietic stem cells differentially regulate long-term hematopoietic output. Proc Natl Acad Sci U S A. 2010;107(32):14235-40. doi:10.1073/pnas.1009798107

12. Lam J, van den Bosch M, Wegrzyn J, Parker J, Ibrahim R, Slowski K, et al. miR-143/145 differentially regulate hematopoietic stem and progenitor activity through suppression of canonical TGFbeta signaling. Nat Commun. 2018;9(1):2418. doi:10.1038/s41467-018-04831-3

13. Wunsche P, Eckert ESP, Holland-Letz T, Paruzynski A, Hotz-Wagenblatt A, Fronza R, et al. Mapping Active Gene-Regulatory Regions in Human Repopulating Long-Term HSCs. Cell Stem Cell. 2018;23(1):132-46 e9. doi:10.1016/j.stem.2018.06.003

14. Thiele S, Wittmann J, Jack HM, Pahl A. miR-9 enhances IL-2 production in activated human CD4(+) T cells by repressing Blimp-1. Eur J Immunol. 2012;42(8):2100-8. doi:10.1002/eji.201142203

15. Ghisi M, Corradin A, Basso K, Frasson C, Serafin V, Mukherjee S, et al. Modulation of microRNA expression in human T-cell development: targeting of NOTCH3 by miR-150. Blood. 2011;117(26):7053-62. doi:10.1182/blood-2010-12-326629

16. Haetscher N, Feuermann Y, Wingert S, Rehage M, Thalheimer FB, Weiser C, et al. STAT5-regulated microRNA-193b controls haematopoietic stem and progenitor cell expansion by modulating cytokine receptor signalling. Nat Commun. 2015;6:8928. doi:10.1038/ncomms9928

17. Belkaya S, Murray SE, Eitson JL, de la Morena MT, Forman JA, van Oers NSC. Transgenic expression of microRNA-185 causes a developmental arrest of T cells by targeting multiple genes including Mzb1. J Biol Chem. 2013;288(42):30752-62. doi:10.1074/jbc.M113.503532

18. Li Z, Zhang S, Wan Y, Cai M, Wang W, Zhu Y, et al. MicroRNA-146a Overexpression Impairs the Positive Selection during T Cell Development. Front Immunol. 2017;8:2006. doi:10.3389/fimmu.2017.02006

19. Kurkewich JL, Boucher A, Klopfenstein N, Baskar R, Kapur R, Dahl R. The mirn23a and mirn23b microrna clusters are necessary for proper hematopoietic progenitor cell production and differentiation. Exp Hematol. 2018;59:14-29. doi:10.1016/j.exphem.2017.12.007

20. Xue Q, Guo ZY, Li W, Wen WH, Meng YL, Jia LT, et al. Human activated CD4(+) T lymphocytes increase IL-2 expression by downregulating microRNA-181c. Mol Immunol. 2011;48(4):592-9. doi:10.1016/j.molimm.2010.10.021

21. Li QJ, Chau J, Ebert PJ, Sylvester G, Min H, Liu G, et al. miR-181a is an intrinsic modulator of T cell sensitivity and selection. Cell. 2007;129(1):147-61. doi:10.1016/j.cell.2007.03.008

22. Crisafulli L, Muggeo S, Uva P, Wang Y, Iwasaki M, Locatelli S, et al. MicroRNA-127-3p controls murine hematopoietic stem cell maintenance by limiting differentiation. Haematologica. 2019;104(9):1744-55. doi:10.3324/haematol.2018.198499

23. Cheng G, Yu A, Malek TR. T-cell tolerance and the multi-functional role of IL-2R signaling in T-regulatory cells. Immunol Rev. 2011;241(1):63-76. doi:10.1111/j.1600-065X.2011.01004.x

24. Mitsumura T, Ito Y, Chiba T, Matsushima T, Kurimoto R, Tanaka Y, et al. Ablation of miR-146b in mice causes hematopoietic malignancy. Blood Adv. 2018;2(23):3483-91. doi:10.1182/bloodadvances.2018017954

25. Hu L, Mao L, Liu S, Zhao J, Chen C, Guo M, et al. Functional Role of MicroRNAs in Thymocyte Development. Int Arch Allergy Immunol. 2019;178(4):315-22. doi:10.1159/000496093

26. Popovic R, Riesbeck LE, Velu CS, Chaubey A, Zhang J, Achille NJ, et al. Regulation of mir-196b by MLL and its overexpression by MLL fusions contributes to immortalization. Blood. 2009;113(14):3314-22. doi:10.1182/blood-2008-04-154310

27. O'Connell RM, Rao DS, Chaudhuri AA, Baltimore D. Physiological and pathological roles for microRNAs in the immune system. Nat Rev Immunol. 2010;10(2):111-22. doi:10.1038/nri2708

28. Mildner A, Chapnik E, Varol D, Aychek T, Lampl N, Rivkin N, et al. MicroRNA-142 controls thymocyte proliferation. Eur J Immunol. 2017;47(7):1142-52. doi:10.1002/eji.201746987

29. Xiao C, Srinivasan L, Calado DP, Patterson HC, Zhang B, Wang J, et al. Lymphoproliferative disease and autoimmunity in mice with increased miR-17-92 expression in lymphocytes. Nat Immunol. 2008;9(4):405-14. doi:10.1038/ni1575

30. Baltimore D, Boldin MP, O'Connell RM, Rao DS, Taganov KD. MicroRNAs: new regulators of immune cell development and function. Nat Immunol. 2008;9(8):839-45. doi:10.1038/ni.f.209

31. Cobb BS, Nesterova TB, Thompson E, Hertweck A, O'Connor E, Godwin J, et al. T cell lineage choice and differentiation in the absence of the RNase III enzyme Dicer. J Exp Med. 2005;201(9):1367-73. doi:10.1084/jem.20050572

32. Xiao C, Calado DP, Galler G, Thai TH, Patterson HC, Wang J, et al. MiR-150 controls B cell differentiation by targeting the transcription factor c-Myb. Cell. 2007;131(1):146-59. doi:10.1016/j.cell.2007.07.021

33. Muljo SA, Ansel KM, Kanellopoulou C, Livingston DM, Rao A, Rajewsky K. Aberrant T cell differentiation in the absence of Dicer. J Exp Med. 2005;202(2):261-9. doi:10.1084/jem.20050678

34. Kurkewich JL, Hansen J, Klopfenstein N, Zhang H, Wood C, Boucher A, et al. The miR-23a~27a~24-2 microRNA cluster buffers transcription and signaling pathways during hematopoiesis. PLoS Genet. 2017;13(7):e1006887. doi:10.1371/journal.pgen.1006887

35. Belkaya S, van Oers NS. Transgenic expression of microRNA-181d augments the stress-sensitivity of CD4(+)CD8(+) thymocytes. PLoS One. 2014;9(1):e85274. doi:10.1371/journal.pone.0085274

36. Wu R, Zeng J, Yuan J, Deng X, Huang Y, Chen L, et al. MicroRNA-210 overexpression promotes psoriasis-like inflammation by inducing Th1 and Th17 cell differentiation. J Clin Invest. 2018;128(6):2551-68. doi:10.1172/JCI97426

37. Shi Y, Dai S, Qiu C, Wang T, Zhou Y, Xue C, et al. MicroRNA-219a-5p suppresses intestinal inflammation through inhibiting Th1/Th17-mediated immune responses in inflammatory bowel disease. Mucosal Immunol. 2020;13(2):303-12. doi:10.1038/s41385-019-0216-7

38. Kohlhaas S, Garden OA, Scudamore C, Turner M, Okkenhaug K, Vigorito E. Cutting edge: the Foxp3 target miR-155 contributes to the development of regulatory T cells. J Immunol. 2009;182(5):2578-82. doi:10.4049/jimmunol.0803162

39. Banerjee A, Schambach F, DeJong CS, Hammond SM, Reiner SL. Micro-RNA-155 inhibits IFN-gamma signaling in CD4+ T cells. Eur J Immunol. 2010;40(1):225-31. doi:10.1002/eji.200939381

40. Thai TH, Calado DP, Casola S, Ansel KM, Xiao C, Xue Y, et al. Regulation of the germinal center response by microRNA-155. Science. 2007;316(5824):604-8. doi:10.1126/science.1141229

41. Hu R, Kagele DA, Huffaker TB, Runtsch MC, Alexander M, Liu J, et al. miR-155 promotes T follicular helper cell accumulation during chronic, low-grade inflammation. Immunity. 2014;41(4):605-19. doi:10.1016/j.immuni.2014.09.015

42. Takahashi H, Kanno T, Nakayamada S, Hirahara K, Sciume G, Muljo SA, et al. TGF-beta and retinoic acid induce the microRNA miR-10a, which targets Bcl-6 and constrains the plasticity of helper T cells. Nat Immunol. 2012;13(6):587-95. doi:10.1038/ni.2286

43. Wu W, He C, Liu C, Cao AT, Xue X, Evans-Marin HL, et al. miR-10a inhibits dendritic cell activation and Th1/Th17 cell immune responses in IBD. Gut. 2015;64(11):1755-64. doi:10.1136/gutjnl-2014-307980

44. Stittrich AB, Haftmann C, Sgouroudis E, Kuhl AA, Hegazy AN, Panse I, et al. The microRNA miR-182 is induced by IL-2 and promotes clonal expansion of activated helper T lymphocytes. Nat Immunol. 2010;11(11):1057-62. doi:10.1038/ni.1945

45. Guan H, Singh UP, Rao R, Mrelashvili D, Sen S, Hao H, et al. Inverse correlation of expression of microRNA-140-5p with progression of multiple sclerosis and differentiation of encephalitogenic T helper type 1 cells. Immunology. 2016;147(4):488-98. doi:10.1111/imm.12583

46. Chen X, Zhang XL, Zhang GH, Gao YF. Artesunate promotes Th1 differentiation from CD4+ T cells to enhance cell apoptosis in ovarian cancer via miR-142. Braz J Med Biol Res. 2019;52(5):e7992. doi:10.1590/1414-431X20197992

47. Steiner DF, Thomas MF, Hu JK, Yang Z, Babiarz JE, Allen CD, et al. MicroRNA-29 regulates T-box transcription factors and interferon-gamma production in helper T cells. Immunity. 2011;35(2):169-81. doi:10.1016/j.immuni.2011.07.009

48. Fujiwara M, Raheja R, Garo LP, Ajay AK, Kadowaki-Saga R, Karandikar SH, et al. microRNA-92a promotes CNS autoimmunity by modulating the regulatory and inflammatory T cell balance. J Clin Invest. 2022;132(10). doi:10.1172/JCI155693

49. Maddalon A, Iulini M, Galbiati V, Colosio C, Mandic-Rajcevic S, Corsini E. Direct Effects of Glyphosate on In Vitro T Helper Cell Differentiation and Cytokine Production. Front Immunol. 2022;13:854837. doi:10.3389/fimmu.2022.854837

50. Zhang Y, Liu W, Chen Y, Liu J, Wu K, Su L, et al. A Cellular MicroRNA Facilitates Regulatory T Lymphocyte Development by Targeting the FOXP3 Promoter TATA-Box Motif. J Immunol. 2018;200(3):1053-63. doi:10.4049/jimmunol.1700196

51. Lazarevic V, Glimcher LH. T-bet in disease. Nat Immunol. 2011;12(7):597-606. doi:10.1038/ni.2059

52. Rossi RL, Rossetti G, Wenandy L, Curti S, Ripamonti A, Bonnal RJ, et al. Distinct microRNA signatures in human lymphocyte subsets and enforcement of the naive state in CD4+ T cells by the microRNA miR-125b. Nat Immunol. 2011;12(8):796-803. doi:10.1038/ni.2057

53. Jiang S, Li C, Olive V, Lykken E, Feng F, Sevilla J, et al. Molecular dissection of the miR-17-92 cluster's critical dual roles in promoting Th1 responses and preventing inducible Treg differentiation. Blood. 2011;118(20):5487-97. doi:10.1182/blood-2011-05-355644

54. Liu SQ, Jiang S, Li C, Zhang B, Li QJ. miR-17-92 cluster targets phosphatase and tensin homology and Ikaros Family Zinc Finger 4 to promote TH17-mediated inflammation. J Biol Chem. 2014;289(18):12446-56. doi:10.1074/jbc.M114.550723

55. Wu T, Wieland A, Araki K, Davis CW, Ye L, Hale JS, et al. Temporal expression of microRNA cluster miR-17-92 regulates effector and memory CD8+ T-cell differentiation. Proc Natl Acad Sci U S A. 2012;109(25):9965-70. doi:10.1073/pnas.1207327109

56. Du J, Gao R, Wang Y, Nguyen T, Yang F, Shi Y, et al. MicroRNA-26a/b have protective roles in oral lichen planus. Cell Death Dis. 2020;11(1):15. doi:10.1038/s41419-019-2207-8

57. Cotrim-Sousa L, Freire-Assis A, Pezzi N, Tanaka PP, Oliveira EH, Passos GA. Adhesion between medullary thymic epithelial cells and thymocytes is regulated by miR-181b-5p and miR-30b. Mol Immunol. 2019;114:600-11. doi:10.1016/j.molimm.2019.09.010

58. Yang L, Boldin MP, Yu Y, Liu CS, Ea CK, Ramakrishnan P, et al. miR-146a controls the resolution of T cell responses in mice. J Exp Med. 2012;209(9):1655-70. doi:10.1084/jem.20112218

59. Zhao H, Li G, Wang R, Tao Z, Ma Q, Zhang S, et al. Silencing of microRNA-494 inhibits the neurotoxic Th1 shift via regulating HDAC2-STAT4 cascade in ischaemic stroke. Br J Pharmacol. 2020;177(1):128-44. doi:10.1111/bph.14852

60. Wan C, Bi W, Lin P, Zhang Y, Tian J, Fang S, et al. MicroRNA 182 promotes T helper 1 cell by repressing hypoxia induced factor 1 alpha in experimental autoimmune encephalomyelitis. Eur J Immunol. 2019;49(12):2184-94. doi:10.1002/eji.201948111

61. Chong MM, Rasmussen JP, Rudensky AY, Littman DR. The RNAseIII enzyme Drosha is critical in T cells for preventing lethal inflammatory disease. J Exp Med. 2008;205(9):2005-17. doi:10.1084/jem.20081219

62. Zhu D, Tian J, Wu X, Li M, Tang X, Rui K, et al. G-MDSC-derived exosomes attenuate collagen-induced arthritis by impairing Th1 and Th17 cell responses. Biochim Biophys Acta Mol Basis Dis. 2019;1865(12):165540. doi:10.1016/j.bbadis.2019.165540

63. Bronevetsky Y, Villarino AV, Eisley CJ, Barbeau R, Barczak AJ, Heinz GA, et al. T cell activation induces proteasomal degradation of Argonaute and rapid remodeling of the microRNA repertoire. J Exp Med. 2013;210(2):417-32. doi:10.1084/jem.20111717

64. Ge Y, Sun M, Wu W, Ma C, Zhang C, He C, et al. MicroRNA-125a suppresses intestinal mucosal inflammation through targeting ETS-1 in patients with inflammatory bowel diseases. J Autoimmun. 2019;101:109-20. doi:10.1016/j.jaut.2019.04.014

65. Cho S, Wu CJ, Nguyen DT, Lin LL, Chen MC, Khan AA, et al. A Novel miR-24-TCF1 Axis in Modulating Effector T Cell Responses. J Immunol. 2017;198(10):3919-26. doi:10.4049/jimmunol.1601404

66. Okoye IS, Coomes SM, Pelly VS, Czieso S, Papayannopoulos V, Tolmachova T, et al. MicroRNA-containing T-regulatory-cell-derived exosomes suppress pathogenic T helper 1 cells. Immunity. 2014;41(1):89-103. doi:10.1016/j.immuni.2014.05.019

67. Kim M, Lee SH, Kim Y, Kwon Y, Park Y, Lee HK, et al. Human Adipose Tissue-Derived Mesenchymal Stem Cells Attenuate Atopic Dermatitis by Regulating the Expression of MIP-2, miR-122a-SOCS1 Axis, and Th1/Th2 Responses. Front Pharmacol. 2018;9:1175. doi:10.3389/fphar.2018.01175

68. Haftmann C, Stittrich AB, Zimmermann J, Fang Z, Hradilkova K, Bardua M, et al. miR-148a is upregulated by Twist1 and T-bet and promotes Th1-cell survival by regulating the proapoptotic gene Bim. Eur J Immunol. 2015;45(4):1192-205. doi:10.1002/eji.201444633

69. Gu Y, Zhou H, Yu H, Yang W, Wang B, Qian F, et al. miR-99a regulates CD4(+) T cell differentiation and attenuates experimental autoimmune encephalomyelitis by mTOR-mediated glycolysis. Mol Ther Nucleic Acids. 2021;26:1173-85. doi:10.1016/j.omtn.2021.07.010

70. Du X, Zhu M, Zhang T, Wang C, Tao J, Yang S, et al. The Recombinant Eg.P29-Mediated miR-126a-5p Promotes the Differentiation of Mouse Naive CD4(+) T Cells via DLK1-Mediated Notch1 Signal Pathway. Front Immunol. 2022;13:773276. doi:10.3389/fimmu.2022.773276

71. He S, Zhou J, Ma Y, Wang W, Yang J. MicroRNA-19a Inhibition Directly and Indirectly Ameliorates Th2 Airway Inflammation in Asthma by Targeting RUNX3. Inflammation. 2023;46(1):370-87. doi:10.1007/s10753-022-01739-5

72. Li D, Du X, Zhu M, Yang S, Zhao W. MiR-374b-5p Regulates T Cell Differentiation and Is Associated with rEg.P29 Immunity. Biomed Res Int. 2020;2020:8024763. doi:10.1155/2020/8024763

73. Wang J, Liu X, Hao C, Lu Y, Duan X, Liang R, et al. MEG3 modulates TIGIT expression and CD4 + T cell activation through absorbing miR-23a. Mol Cell Biochem. 2019;454(1-2):67-76. doi:10.1007/s11010-018-3453-2

74. Li H, Wang Y, Han X. ESP-B4 promotes nasal epithelial cell-derived extracellular vesicles containing miR-146a-5p to modulate Smad3/GATA-3 thus relieving allergic rhinitis: ESP-B4/miR-146a-5p in AR. Phytomedicine. 2023;108:154516. doi:10.1016/j.phymed.2022.154516

75. Liu J, Jiang Y, Han M, Jiang L, Liang D, Li S, et al. MicroRNA-345-5p acts as an anti-inflammatory regulator in experimental allergic rhinitis via the TLR4/NF-kappaB pathway. Int Immunopharmacol. 2020;86:106522. doi:10.1016/j.intimp.2020.106522

76. Jiang M, Fang H, Dang E, Zhang J, Qiao P, Yu C, et al. Small Extracellular Vesicles Containing miR-381-3p from Keratinocytes Promote T Helper Type 1 and T Helper Type 17 Polarization in Psoriasis. J Invest Dermatol. 2021;141(3):563-74. doi:10.1016/j.jid.2020.07.009

77. Qin Z, Wang PY, Wan JJ, Zhang Y, Wei J, Sun Y, et al. MicroRNA124-IL6R Mediates the Effect of Nicotine in Inflammatory Bowel Disease by Shifting Th1/Th2 Balance Toward Th1. Front Immunol. 2020;11:235. doi:10.3389/fimmu.2020.00235

78. Sun J, Wu M, Wang L, Wang P, Xiao T, Wang S, et al. miRNA-21, which disrupts metabolic reprogramming to facilitate CD4(+) T cell polarization toward the Th2 phenotype, accelerates arsenite-induced hepatic fibrosis. Ecotoxicol Environ Saf. 2022;248:114321. doi:10.1016/j.ecoenv.2022.114321

79. Zhou J, Lu Y, Wu W, Feng Y. HMSC-Derived Exosome Inhibited Th2 Cell Differentiation via Regulating miR-146a-5p/SERPINB2 Pathway. J Immunol Res. 2021;2021:6696525. doi:10.1155/2021/6696525

80. Kim HJ, Park SO, Byeon HW, Eo JC, Choi JY, Tanveer M, et al. T cell-intrinsic miR-155 is required for Th2 and Th17-biased responses in acute and chronic airway inflammation by targeting several different transcription factors. Immunology. 2022;166(3):357-79. doi:10.1111/imm.13477

81. Matsuyama H, Suzuki HI, Nishimori H, Noguchi M, Yao T, Komatsu N, et al. miR-135b mediates NPM-ALK-driven oncogenicity and renders IL-17-producing immunophenotype to anaplastic large cell lymphoma. Blood. 2011;118(26):6881-92. doi:10.1182/blood-2011-05-354654

82. Kilic A, Santolini M, Nakano T, Schiller M, Teranishi M, Gellert P, et al. A systems immunology approach identifies the collective impact of 5 miRs in Th2 inflammation. JCI Insight. 2018;3(11). doi:10.1172/jci.insight.97503

83. Sun H, Wang T, Zhang W, Dong H, Gu W, Huang L, et al. LncRNATUG1 Facilitates Th2 Cell Differentiation by Targeting the miR-29c/B7-H3 Axis on Macrophages. Front Immunol. 2021;12:631450. doi:10.3389/fimmu.2021.631450

84. Mu Y, Zhang J, Liu Y, Ma J, Jiang D, Zhang X, et al. CD226 deficiency on regulatory T cells aggravates renal fibrosis via up-regulation of Th2 cytokines through miR-340. J Leukoc Biol. 2020;107(4):573-87. doi:10.1002/JLB.2MA1119-174RR

85. Gu W, Li G, Zhang W, Zhang X, He Y, Huang L, et al. MiR-29b regulates Th2 cell differentiation in asthma by targeting inducible B7-H3 and STAT3. Clin Transl Allergy. 2022;12(1):e12114. doi:10.1002/clt2.12114

86. Wang T, Zhou Q, Shang Y. Downregulation of miRNA-451a Promotes the Differentiation of CD4+ T Cells towards Th2 Cells by Upregulating ETS1 in Childhood Asthma. J Innate Immun. 2021;13(1):38-48. doi:10.1159/000509714

87. Chen Z, Deng Y, Li F, Xiao B, Zhou X, Tao Z. MicroRNA-466a-3p attenuates allergic nasal inflammation in mice by targeting GATA3. Clin Exp Immunol. 2019;197(3):366-75. doi:10.1111/cei.13312

88. Wu XH, Zhao SJ, Huang WQ, Huang LH, Luo XY, Long SL. Long non-coding RNA MALAT1 promotes Th2 differentiation by regulating microRNA-135b-5p/GATA-3 axis in children with allergic rhinitis. Kaohsiung J Med Sci. 2022;38(10):971-80. doi:10.1002/kjm2.12587

89. Zhu X, Wang X, Wang Y, Zhao Y. The regulatory network among CircHIPK3, LncGAS5, and miR-495 promotes Th2 differentiation in allergic rhinitis. Cell Death Dis. 2020;11(4):216. doi:10.1038/s41419-020-2394-3

90. Liu Q, Shen Y, Xiao Y, Xiang H, Chu L, Wang T, et al. Increased miR-124-3p alleviates type 2 inflammatory response in allergic rhinitis via IL-4Ralpha. Inflamm Res. 2022;71(10-11):1271-82. doi:10.1007/s00011-022-01614-x

91. Hur J, Rhee CK, Lee SY, Kim YK, Kang JY. MicroRNA-21 inhibition attenuates airway inflammation and remodelling by modulating the transforming growth factor beta-Smad7 pathway. Korean J Intern Med. 2021;36(3):706-20. doi:10.3904/kjim.2020.132

92. Guo C, Liu J, Hao P, Wang Y, Sui S, Li L, et al. The Potential Inhibitory Effects of miR-19b on Ocular Inflammation are Mediated Upstream of the JAK/STAT Pathway in a Murine Model of Allergic Conjunctivitis. Invest Ophthalmol Vis Sci. 2020;61(3):8. doi:10.1167/iovs.61.3.8

93. Pua HH, Steiner DF, Patel S, Gonzalez JR, Ortiz-Carpena JF, Kageyama R, et al. MicroRNAs 24 and 27 Suppress Allergic Inflammation and Target a Network of Regulators of T Helper 2 Cell-Associated Cytokine Production. Immunity. 2016;44(4):821-32. doi:10.1016/j.immuni.2016.01.003

94. Liu L, Liu Y, Yuan M, Xu L, Sun H. Elevated expression of microRNA-873 facilitates Th17 differentiation by targeting forkhead box O1 (Foxo1) in the pathogenesis of systemic lupus erythematosus. Biochem Biophys Res Commun. 2017;492(3):453-60. doi:10.1016/j.bbrc.2017.08.075

95. Huo J, Liu T, Li F, Song X, Hou X. MicroRNA‑21‑5p protects melanocytes via targeting STAT3 and modulating Treg/Teff balance to alleviate vitiligo. Mol Med Rep. 2021;23(1). doi:10.3892/mmr.2020.11689

96. Wu T, Lei Y, Jin S, Zhao Q, Cheng W, Xi Y, et al. miRNA-467b inhibits Th17 differentiation by targeting eIF4E in experimental autoimmune encephalomyelitis. Mol Immunol. 2021;133:23-33. doi:10.1016/j.molimm.2021.02.008

97. Yang X, He Q, Guo Z, Xiong F, Li Y, Pan Y, et al. MicroRNA-425 facilitates pathogenic Th17 cell differentiation by targeting forkhead box O1 (Foxo1) and is associated with inflammatory bowel disease. Biochem Biophys Res Commun. 2018;496(2):352-8. doi:10.1016/j.bbrc.2018.01.055

98. Ji Q, Liu J, Dong Y, Wang L, Dong K, Setiz B, et al. Exosomes derived from thymic stromal lymphopoietin-treated dendritic cells regulate T helper 17/regulatory T cell differentiation via miR-21/Smad7 axis. Exp Cell Res. 2021;398(1):112393. doi:10.1016/j.yexcr.2020.112393

99. Wu R, Li X, Li S, Tang G, Zhang S, Zhu Y, et al. Decreased microRNA-126 expression in psoriatic CD4(+) T cells promotes T-helper 17 cell differentiation and the formation of dermatitis in imiquimod-induced psoriasis-like mice. J Dermatol. 2022;49(4):432-40. doi:10.1111/1346-8138.16272

100. Yao X, Wang Q, Zeng P, Hou L, Yang Y, Lu D, et al. LncRNA HOTTIP from synovial fibroblast-derived exosomes: A novel molecular target for rheumatoid arthritis through the miR-1908-5p/STAT3 axis. Exp Cell Res. 2021;409(2):112943. doi:10.1016/j.yexcr.2021.112943

101. Xuan J, Huang A, Hu D, Geng J, Tian Y, Cheng Z, et al. Huagan tongluo Fang improves liver fibrosis via down-regulating miR-184 and up-regulating FOXO1 to inhibit Th17 cell differentiation. Exp Mol Pathol. 2020;115:104447. doi:10.1016/j.yexmp.2020.104447

102. Xie M, Wang J, Gong W, Xu H, Pan X, Chen Y, et al. NF-kappaB-driven miR-34a impairs Treg/Th17 balance via targeting Foxp3. J Autoimmun. 2019;102:96-113. doi:10.1016/j.jaut.2019.04.018

103. Geng L, Tang X, Wang S, Sun Y, Wang D, Tsao BP, et al. Reduced Let-7f in Bone Marrow-Derived Mesenchymal Stem Cells Triggers Treg/Th17 Imbalance in Patients With Systemic Lupus Erythematosus. Front Immunol. 2020;11:233. doi:10.3389/fimmu.2020.00233

104. Li ZH, Wang YF, He DD, Zhang XM, Zhou YL, Yue H, et al. Let-7f-5p suppresses Th17 differentiation via targeting STAT3 in multiple sclerosis. Aging (Albany NY). 2019;11(13):4463-77. doi:10.18632/aging.102093

105. Ichiyama K, Gonzalez-Martin A, Kim BS, Jin HY, Jin W, Xu W, et al. The MicroRNA-183-96-182 Cluster Promotes T Helper 17 Cell Pathogenicity by Negatively Regulating Transcription Factor Foxo1 Expression. Immunity. 2016;44(6):1284-98. doi:10.1016/j.immuni.2016.05.015

106. Busbee PB, Bam M, Yang X, Abdulla OA, Zhou J, Ginsberg JPJ, et al. Dysregulated TP53 Among PTSD Patients Leads to Downregulation of miRNA let-7a and Promotes an Inflammatory Th17 Phenotype. Front Immunol. 2021;12:815840. doi:10.3389/fimmu.2021.815840

107. Yang R, Huang H, Cui S, Zhou Y, Zhang T, Zhou Y. IFN-gamma promoted exosomes from mesenchymal stem cells to attenuate colitis via miR-125a and miR-125b. Cell Death Dis. 2020;11(7):603. doi:10.1038/s41419-020-02788-0

108. Botta C, Cuce M, Pitari MR, Caracciolo D, Gulla A, Morelli E, et al. MiR-29b antagonizes the pro-inflammatory tumor-promoting activity of multiple myeloma-educated dendritic cells. Leukemia. 2018;32(4):1003-15. doi:10.1038/leu.2017.336

109. Li J, Xia Y, Fan X, Wu X, Yang F, Hu S, et al. Extracellular vesicles derived from miR-199a-5p-modified adipose-derived mesenchymal stem cells alleviate immune thrombocytopenia by inhibiting T helper 17 differentiation. Lab Invest. 2021;101(3):318-27. doi:10.1038/s41374-020-00515-z

110. Ye X, Lu Q, Yang A, Rao J, Xie W, He C, et al. MiR-206 regulates the Th17/Treg ratio during osteoarthritis. Mol Med. 2021;27(1):64. doi:10.1186/s10020-021-00315-1

111. Qiu YY, Wu Y, Lin MJ, Bian T, Xiao YL, Qin C. LncRNA-MEG3 functions as a competing endogenous RNA to regulate Treg/Th17 balance in patients with asthma by targeting microRNA-17/ RORgammat. Biomed Pharmacother. 2019;111:386-94. doi:10.1016/j.biopha.2018.12.080

112. Gu D, Nan Q, Miao Y, Yang H, Li M, Ye Y, et al. KT2 alleviates ulcerative colitis by reducing Th17 cell differentiation through the miR-302c-5p/STAT3 axis. Eur J Cell Biol. 2022;101(2):151223. doi:10.1016/j.ejcb.2022.151223

113. Sun R, Zhang PP, Weng XQ, Gao XD, Huang CX, Wang L, et al. Therapeutic targeting miR130b counteracts diffuse large B-cell lymphoma progression via OX40/OX40L-mediated interaction with Th17 cells. Signal Transduct Target Ther. 2022;7(1):80. doi:10.1038/s41392-022-00895-2

114. Rossi M, Altomare E, Botta C, Gallo Cantafio ME, Sarvide S, Caracciolo D, et al. miR-21 antagonism abrogates Th17 tumor promoting functions in multiple myeloma. Leukemia. 2021;35(3):823-34. doi:10.1038/s41375-020-0947-1

115. Zhu E, Wang X, Zheng B, Wang Q, Hao J, Chen S, et al. miR-20b suppresses Th17 differentiation and the pathogenesis of experimental autoimmune encephalomyelitis by targeting RORgammat and STAT3. J Immunol. 2014;192(12):5599-609. doi:10.4049/jimmunol.1303488

116. Liu F, Bu Z, Zhao F, Xiao D. Increased T-helper 17 cell differentiation mediated by exosome-mediated microRNA-451 redistribution in gastric cancer infiltrated T cells. Cancer Sci. 2018;109(1):65-73. doi:10.1111/cas.13429

117. Liu X, Luo M, Meng H, Zeng Q, Xu L, Hu B, et al. MiR-181a regulates CD4(+) T cell activation and differentiation by targeting IL-2 in the pathogenesis of myasthenia gravis. Eur J Immunol. 2019. doi:10.1002/eji.201848007

118. Shi L, Guo H, Li Z, Wang Y, Wang Y, Cui Y. Adenovirus-mediated down-regulation of miR-21-5p alleviates experimental autoimmune uveoretinitis in mice. Int Immunopharmacol. 2019;74:105698. doi:10.1016/j.intimp.2019.105698

119. Di Y, Zhang M, Chen Y, Sun R, Shen M, Tian F, et al. Catalpol Inhibits Tregs-to-Th17 Cell Transdifferentiation by Up-Regulating Let-7g-5p to Reduce STAT3 Protein Levels. Yonsei Med J. 2022;63(1):56-65. doi:10.3349/ymj.2022.63.1.56

120. Cao Y, Wang Z, Yan Y, Ji L, He J, Xuan B, et al. Enterotoxigenic Bacteroidesfragilis Promotes Intestinal Inflammation and Malignancy by Inhibiting Exosome-Packaged miR-149-3p. Gastroenterology. 2021;161(5):1552-66 e12. doi:10.1053/j.gastro.2021.08.003

121. Shi QZ, Yu HM, Chen HM, Liu M, Cheng X. Exosomes derived from mesenchymal stem cells regulate Treg/Th17 balance in aplastic anemia by transferring miR-23a-3p. Clin Exp Med. 2021;21(3):429-37. doi:10.1007/s10238-021-00701-3

122. Wang L, Wang E, Wang Y, Mines R, Xiang K, Sun Z, et al. miR-34a is a microRNA safeguard for Citrobacter-induced inflammatory colon oncogenesis. Elife. 2018;7. doi:10.7554/eLife.39479

123. Zhang Y, Wang ZC, Zhang ZS, Chen F. MicroRNA-155 regulates cervical cancer via inducing Th17/Treg imbalance. Eur Rev Med Pharmacol Sci. 2018;22(12):3719-26. doi:10.26355/eurrev_201806_15251

124. Sun X, Zheng X, Zhang X, Zhang Y, Luo G. Exosomal microRNA-23b-3p from bone marrow mesenchymal stem cells maintains T helper/Treg balance by downregulating the PI3k/Akt/NF-kappaB signaling pathway in intracranial aneurysm. Brain Res Bull. 2020;165:305-15. doi:10.1016/j.brainresbull.2020.09.003

125. Wei Y, Chen S, Sun D, Li X, Wei R, Li X, et al. miR-223-3p promotes autoreactive T(h)17 cell responses in experimental autoimmune uveitis (EAU) by inhibiting transcription factor FOXO3 expression. FASEB J. 2019;33(12):13951-65. doi:10.1096/fj.201901446R

126. Wu M, Sun J, Wang L, Wang P, Xiao T, Wang S, et al. The lncRNA HOTAIR via miR-17-5p is involved in arsenite-induced hepatic fibrosis through regulation of Th17 cell differentiation. J Hazard Mater. 2023;443(Pt B):130276. doi:10.1016/j.jhazmat.2022.130276

127. Yao J, Gao R, Luo M, Li D, Guo L, Yu Z, et al. miR-802 participates in the inflammatory process of inflammatory bowel disease by suppressing SOCS5. Biosci Rep. 2020;40(4). doi:10.1042/BSR20192257

128. Singh NP, Singh UP, Rouse M, Zhang J, Chatterjee S, Nagarkatti PS, et al. Dietary Indoles Suppress Delayed-Type Hypersensitivity by Inducing a Switch from Proinflammatory Th17 Cells to Anti-Inflammatory Regulatory T Cells through Regulation of MicroRNA. J Immunol. 2016;196(3):1108-22. doi:10.4049/jimmunol.1501727

129. Kang L, Zhang X, Ji L, Kou T, Smith SM, Zhao B, et al. The colonic macrophage transcription factor RBP-J orchestrates intestinal immunity against bacterial pathogens. J Exp Med. 2020;217(4). doi:10.1084/jem.20190762

130. He C, Shi Y, Wu R, Sun M, Fang L, Wu W, et al. miR-301a promotes intestinal mucosal inflammation through induction of IL-17A and TNF-alpha in IBD. Gut. 2016;65(12):1938-50. doi:10.1136/gutjnl-2015-309389

131. You G, Cao H, Yan L, He P, Wang Y, Liu B, et al. MicroRNA-10a-3p mediates Th17/Treg cell balance and improves renal injury by inhibiting REG3A in lupus nephritis. Int Immunopharmacol. 2020;88:106891. doi:10.1016/j.intimp.2020.106891

132. Lin J, Tang J, Lin J, He Y, Yu Z, Jiang R, et al. YY1 regulation by miR-124-3p promotes Th17 cell pathogenicity through interaction with T-bet in rheumatoid arthritis. JCI Insight. 2021;6(22). doi:10.1172/jci.insight.149985

133. Sun H, Guo F, Xu L. Downregulation of microRNA-101-3p participates in systemic lupus erythematosus progression via negatively regulating HDAC9. J Cell Biochem. 2020;121(10):4310-20. doi:10.1002/jcb.29624

134. Hu R, Lv W, Zhang S, Liu Y, Sun B, Meng Y, et al. Combining miR-23b exposure with mesenchymal stem cell transplantation enhances therapeutic effects on EAE. Immunol Lett. 2021;229:18-26. doi:10.1016/j.imlet.2020.11.007

135. Jiang Q, Wang Q, Tan S, Cai J, Ye X, Su G, et al. Effects of Plasma-Derived Exosomal miRNA-19b-3p on Treg/T Helper 17 Cell Imbalance in Behcet's Uveitis. Invest Ophthalmol Vis Sci. 2023;64(4):28. doi:10.1167/iovs.64.4.28

136. Bian J, Liu R, Fan T, Liao L, Wang S, Geng W, et al. miR-340 Alleviates Psoriasis in Mice through Direct Targeting of IL-17A. J Immunol. 2018;201(5):1412-20. doi:10.4049/jimmunol.1800189

137. Wang MJ, Huang HJ, Xu YY, Vos H, Gulersonmez C, Stigter E, et al. Metabolic rewiring in keratinocytes by miR-31-5p identifies therapeutic intervention for psoriasis. EMBO Mol Med. 2023;15(4):e15674. doi:10.15252/emmm.202215674

138. Schiavinato J, Haddad R, Saldanha-Araujo F, Baiochi J, Araujo AG, Santos Scheucher P, et al. TGF-beta/atRA-induced Tregs express a selected set of microRNAs involved in the repression of transcripts related to Th17 differentiation. Sci Rep. 2017;7(1):3627. doi:10.1038/s41598-017-03456-8

139. Qu X, Zhou J, Wang T, Han J, Ma L, Yu H, et al. MiR-30a inhibits Th17 differentiation and demyelination of EAE mice by targeting the IL-21R. Brain Behav Immun. 2016;57:193-9. doi:10.1016/j.bbi.2016.03.016

140. Bolandi Z, Mokhberian N, Eftekhary M, Sharifi K, Soudi S, Ghanbarian H, et al. Adipose derived mesenchymal stem cell exosomes loaded with miR-10a promote the differentiation of Th17 and Treg from naive CD4(+) T cell. Life Sci. 2020;259:118218. doi:10.1016/j.lfs.2020.118218

141. Al-Ghezi ZZ, Singh N, Mehrpouya-Bahrami P, Busbee PB, Nagarkatti M, Nagarkatti PS. AhR Activation by TCDD (2,3,7,8-Tetrachlorodibenzo-p-dioxin) Attenuates Pertussis Toxin-Induced Inflammatory Responses by Differential Regulation of Tregs and Th17 Cells Through Specific Targeting by microRNA. Front Microbiol. 2019;10:2349. doi:10.3389/fmicb.2019.02349

142. Li L, Ma X, Zhao YF, Zhang C. MiR-1-3p facilitates Th17 differentiation associating with multiple sclerosis via targeting ETS1. Eur Rev Med Pharmacol Sci. 2020;24(12):6881-92. doi:10.26355/eurrev_202006_21678

143. Xie K, Liu L, Chen J, Liu F. Exosomal miR-1246 derived from human umbilical cord blood mesenchymal stem cells attenuates hepatic ischemia reperfusion injury by modulating T helper 17/regulatory T balance. IUBMB Life. 2019;71(12):2020-30. doi:10.1002/iub.2147

144. Hu C, Zhen Y, Ma Z, Zhao L, Wu H, Shu C, et al. Polyamines from myeloid-derived suppressor cells promote Th17 polarization and disease progression. Mol Ther. 2023;31(2):569-84. doi:10.1016/j.ymthe.2022.10.013

145. Zhou J, Li X, Wu X, Zhang T, Zhu Q, Wang X, et al. Exosomes Released from Tumor-Associated Macrophages Transfer miRNAs That Induce a Treg/Th17 Cell Imbalance in Epithelial Ovarian Cancer. Cancer Immunol Res. 2018;6(12):1578-92. doi:10.1158/2326-6066.CIR-17-0479

146. Nakahama T, Hanieh H, Nguyen NT, Chinen I, Ripley B, Millrine D, et al. Aryl hydrocarbon receptor-mediated induction of the microRNA-132/212 cluster promotes interleukin-17-producing T-helper cell differentiation. Proc Natl Acad Sci U S A. 2013;110(29):11964-9. doi:10.1073/pnas.1311087110

147. Chen J, Adamiak W, Huang G, Atasoy U, Rostami A, Yu S. Interaction of RNA-binding protein HuR and miR-466i regulates GM-CSF expression. Sci Rep. 2017;7(1):17233. doi:10.1038/s41598-017-17371-5

148. Chi X, Guo Y, Zhang L, Zhang J, Du Y, Zhao W, et al. Long non-coding RNA GAS5 regulates Th17/Treg imbalance in childhood pneumonia by targeting miR-217/STAT5. Cell Immunol. 2021;364:104357. doi:10.1016/j.cellimm.2021.104357

149. Liu Y, Cui X, Wang S, Liu J, Zhao N, Huang M, et al. Elevated MicroRNA-326 Levels Regulate the IL-23/IL-23R/Th17 Cell Axis in Hashimoto's Thyroiditis by Targeting a Disintegrin and Metalloprotease 17. Thyroid. 2020;30(9):1327-37. doi:10.1089/thy.2019.0552

150. Wang D, Tang M, Zong P, Liu H, Zhang T, Liu Y, et al. MiRNA-155 Regulates the Th17/Treg Ratio by Targeting SOCS1 in Severe Acute Pancreatitis. Front Physiol. 2018;9:686. doi:10.3389/fphys.2018.00686

151. Bahmani L, Baghi M, Peymani M, Javeri A, Ghaedi K. MiR-141-3p and miR-200a-3p are involved in Th17 cell differentiation by negatively regulating RARB expression. Hum Cell. 2021;34(5):1375-87. doi:10.1007/s13577-021-00558-4

152. Nie J, Zhao Q. Lnc-ITSN1-2, Derived From RNA Sequencing, Correlates With Increased Disease Risk, Activity and Promotes CD4(+) T Cell Activation, Proliferation and Th1/Th17 Cell Differentiation by Serving as a ceRNA for IL-23R via Sponging miR-125a in Inflammatory Bowel Disease. Front Immunol. 2020;11:852. doi:10.3389/fimmu.2020.00852

153. Wang J, Zhang Y, Li H, Chen G, Zou Y, Rin K. Immune effects of miRNA and Th17 cells on beta-Lg allergy in dietary milk based on mouse model. Saudi J Biol Sci. 2020;27(12):3442-8. doi:10.1016/j.sjbs.2020.08.028

154. Fan ZD, Cao Q, Huang N, Ma L, Ma HH, Zhang YY, et al. MicroRNA-125b regulates Th17/Treg cell differentiation and is associated with juvenile idiopathic arthritis. World J Pediatr. 2020;16(1):99-110. doi:10.1007/s12519-019-00265-z

155. Zhang J, Guo Y, Sun Y, Chang L, Wang X. Inhibition of microRNA-448 suppresses CD4(+) T cell inflammatory activation via up-regulating suppressor of cytokine signaling 5 in systemic lupus erythematosus. Biochem Biophys Res Commun. 2022;596:88-96. doi:10.1016/j.bbrc.2022.01.097

156. Zhou L, Wang J, Li J, Li T, Chen Y, June RR, et al. 1,25-Dihydroxyvitamin D3 Ameliorates Collagen-Induced Arthritis via Suppression of Th17 Cells Through miR-124 Mediated Inhibition of IL-6 Signaling. Front Immunol. 2019;10:178. doi:10.3389/fimmu.2019.00178

157. Rutman AK, Negi S, Saberi N, Khan K, Tchervenkov J, Paraskevas S. Extracellular Vesicles From Kidney Allografts Express miR-218-5p and Alter Th17/Treg Ratios. Front Immunol. 2022;13:784374. doi:10.3389/fimmu.2022.784374

158. Li D, Liu L, Du X, Ma W, Zhang J, Piao W. MiRNA-374b-5p and miRNA-106a-5p are related to inflammatory bowel disease via regulating IL-10 and STAT3 signaling pathways. BMC Gastroenterol. 2022;22(1):492. doi:10.1186/s12876-022-02533-1

159. Du C, Liu C, Kang J, Zhao G, Ye Z, Huang S, et al. MicroRNA miR-326 regulates TH-17 differentiation and is associated with the pathogenesis of multiple sclerosis. Nat Immunol. 2009;10(12):1252-9. doi:10.1038/ni.1798

160. Zhang Z, Xue Z, Liu Y, Liu H, Guo X, Li Y, et al. MicroRNA-181c promotes Th17 cell differentiation and mediates experimental autoimmune encephalomyelitis. Brain Behav Immun. 2018;70:305-14. doi:10.1016/j.bbi.2018.03.011

161. Wu Y, Mealer C, Schutt S, Wilson CL, Bastian D, Sofi MH, et al. MicroRNA-31 regulates T-cell metabolism via HIF1alpha and promotes chronic GVHD pathogenesis in mice. Blood Adv. 2022;6(10):3036-52. doi:10.1182/bloodadvances.2021005103

162. Zhang R, Tian A, Wang J, Shen X, Qi G, Tang Y. miR26a modulates Th17/T reg balance in the EAE model of multiple sclerosis by targeting IL6. Neuromolecular Med. 2015;17(1):24-34. doi:10.1007/s12017-014-8335-5

163. Chen Z, Ke X, Wang X, Kang H, Hong S. LncRNA JPX contributes to Treg/Th17 imbalance in allergic rhinitis via targeting the miR-378g/CCL5 axis. Immunopharmacol Immunotoxicol. 2022;44(4):519-24. doi:10.1080/08923973.2022.2055566

164. Han J, Liu Y, Zhen F, Yuan W, Zhang W, Song X, et al. STAT3 Regulates miR-384 Transcription During Th17 Polarization. Front Cell Dev Biol. 2019;7:253. doi:10.3389/fcell.2019.00253

165. Qu X, Han J, Zhang Y, Wang Y, Zhou J, Fan H, et al. MiR-384 Regulates the Th17/Treg Ratio during Experimental Autoimmune Encephalomyelitis Pathogenesis. Front Cell Neurosci. 2017;11:88. doi:10.3389/fncel.2017.00088

166. Guan Y, Ma Y, Tang Y, Liu X, Zhao Y, An L. MiRNA-221-5p suppressed the Th17/Treg ratio in asthma via RORgammat/Foxp3 by targeting SOCS1. Allergy Asthma Clin Immunol. 2021;17(1):123. doi:10.1186/s13223-021-00620-8

167. Montoya MM, Maul J, Singh PB, Pua HH, Dahlstrom F, Wu N, et al. A Distinct Inhibitory Function for miR-18a in Th17 Cell Differentiation. J Immunol. 2017;199(2):559-69. doi:10.4049/jimmunol.1700170

168. Liu X, Zhou F, Yang Y, Wang W, Niu L, Zuo D, et al. MiR-409-3p and MiR-1896 co-operatively participate in IL-17-induced inflammatory cytokine production in astrocytes and pathogenesis of EAE mice via targeting SOCS3/STAT3 signaling. Glia. 2019;67(1):101-12. doi:10.1002/glia.23530

169. Mikami Y, Philips RL, Sciume G, Petermann F, Meylan F, Nagashima H, et al. MicroRNA-221 and -222 modulate intestinal inflammatory Th17 cell response as negative feedback regulators downstream of interleukin-23. Immunity. 2021;54(3):514-25 e6. doi:10.1016/j.immuni.2021.02.015

170. Wang X, Xin S, Wang Y, Ju D, Wu Q, Qiu Y, et al. MicroRNA-146a-5p enhances T helper 17 cell differentiation via decreasing a disintegrin and metalloprotease 17 level in primary sjogren's syndrome. Bioengineered. 2021;12(1):310-24. doi:10.1080/21655979.2020.1870321

171. Song Y, Yang H, Jiang K, Wang BM, Lin R. miR-181a regulates Th17 cells distribution via up-regulated BCL-2 in primary biliary cholangitis. Int Immunopharmacol. 2018;64:386-93. doi:10.1016/j.intimp.2018.09.027

172. Zhao C, Li XY, Li ZY, Li M, Liu ZD. Moxibustion regulates T-regulatory/T-helper 17 cell balance by modulating the microRNA-221/suppressor of cytokine signaling 3 axis in a mouse model of rheumatoid arthritis. J Integr Med. 2022;20(5):453-62. doi:10.1016/j.joim.2022.06.002

173. Li JQ, Tian JM, Fan XR, Wang ZY, Ling J, Wu XF, et al. miR-106b-5p induces immune imbalance of Treg/Th17 in immune thrombocytopenic purpura through NR4A3/Foxp3 pathway. Cell Cycle. 2020;19(11):1265-74. doi:10.1080/15384101.2020.1746485

174. Xu QF, Peng HP, Lu XR, Hu Y, Xu ZH, Xu JK. Oleanolic acid regulates the Treg/Th17 imbalance in gastric cancer by targeting IL-6 with miR-98-5p. Cytokine. 2021;148:155656. doi:10.1016/j.cyto.2021.155656

175. Pang B, Zhen Y, Hu C, Ma Z, Lin S, Yi H. Myeloid-derived suppressor cells shift Th17/Treg ratio and promote systemic lupus erythematosus progression through arginase-1/miR-322-5p/TGF-beta pathway. Clin Sci (Lond). 2020;134(16):2209-22. doi:10.1042/CS20200799

176. Bian Z, Lei W, Li Q, Xue W, Gao Y, Zeng Y, et al. Gm15575 functions as a ceRNA to up-regulate CCL7 expression through sponging miR-686 in Th17 cells. Mol Immunol. 2020;125:32-42. doi:10.1016/j.molimm.2020.06.027

177. Zhao M, Sun D, Guan Y, Wang Z, Sang D, Liu M, et al. Disulfiram and Diphenhydramine Hydrochloride Upregulate miR-30a to Suppress IL-17-Associated Autoimmune Inflammation. J Neurosci. 2016;36(35):9253-66. doi:10.1523/JNEUROSCI.4587-15.2016

178. Ding Y, Wang L, Wu H, Zhao Q, Wu S. Exosomes derived from synovial fibroblasts under hypoxia aggravate rheumatoid arthritis by regulating Treg/Th17 balance. Exp Biol Med (Maywood). 2020;245(14):1177-86. doi:10.1177/1535370220934736

179. Mycko MP, Cichalewska M, Machlanska A, Cwiklinska H, Mariasiewicz M, Selmaj KW. MicroRNA-301a regulation of a T-helper 17 immune response controls autoimmune demyelination. Proc Natl Acad Sci U S A. 2012;109(20):E1248-57. doi:10.1073/pnas.1114325109

180. Hua M, Li J, Wang C, Shao L, Hou M, Peng J, et al. Aberrant expression of microRNA in CD4(+) cells contributes to Th17/Treg imbalance in primary immune thrombocytopenia. Thromb Res. 2019;177:70-8. doi:10.1016/j.thromres.2019.03.005

181. Jin S, Sun S, Ling H, Ma J, Zhang X, Xie Z, et al. Protectin DX restores Treg/T(h)17 cell balance in rheumatoid arthritis by inhibiting NLRP3 inflammasome via miR-20a. Cell Death Dis. 2021;12(3):280. doi:10.1038/s41419-021-03562-6

182. Kastle M, Bartel S, Geillinger-Kastle K, Irmler M, Beckers J, Ryffel B, et al. microRNA cluster 106a~363 is involved in T helper 17 cell differentiation. Immunology. 2017;152(3):402-13. doi:10.1111/imm.12775

183. Li T, Li M, Xu C, Xu X, Ding J, Cheng L, et al. miR‑146a regulates the function of Th17 cell differentiation to modulate cervical cancer cell growth and apoptosis through NF‑kappaB signaling by targeting TRAF6. Oncol Rep. 2019;41(5):2897-908. doi:10.3892/or.2019.7046

184. He Y, Ji D, Lu W, Li F, Huang X, Huang R, et al. Bone marrow mesenchymal stem cell-derived exosomes induce the Th17/Treg imbalance in immune thrombocytopenia through miR-146a-5p/IRAK1 axis. Hum Cell. 2021;34(5):1360-74. doi:10.1007/s13577-021-00547-7

185. Chen J, Huang F, Hou Y, Lin X, Liang R, Hu X, et al. TGF-beta-induced CD4+ FoxP3+ regulatory T cell-derived extracellular vesicles modulate Notch1 signaling through miR-449a and prevent collagen-induced arthritis in a murine model. Cell Mol Immunol. 2021;18(11):2516-29. doi:10.1038/s41423-021-00764-y

186. Zhang L, Sun P, Zhang Y, Xu Y, Sun Y. miR-182-5p inhibits the pathogenic Th17 response in experimental autoimmune uveitis mice via suppressing TAF15. Biochem Biophys Res Commun. 2020;529(3):784-92. doi:10.1016/j.bbrc.2020.06.073

187. Zhang MF, Yang P, Shen MY, Wang X, Gao NX, Zhou XP, et al. MicroRNA-26b-5p alleviates murine collagen-induced arthritis by modulating Th17 cell plasticity. Cell Immunol. 2021;365:104382. doi:10.1016/j.cellimm.2021.104382

188. Xue Y, Zhang L, Guo R, Shao X, Shi M, Yuan C, et al. miR-485 regulates Th17 generation and pathogenesis in experimental autoimmune encephalomyelitis through targeting STAT3. J Neuroimmunol. 2023;379:578100. doi:10.1016/j.jneuroim.2023.578100

189. Abdulla OA, Neamah W, Sultan M, Chatterjee S, Singh N, Nagarkatti M, et al. AhR Ligands Differentially Regulate miRNA-132 Which Targets HMGB1 and to Control the Differentiation of Tregs and Th-17 Cells During Delayed-Type Hypersensitivity Response. Front Immunol. 2021;12:635903. doi:10.3389/fimmu.2021.635903

190. Li X, Luo F, Li J, Luo C. MiR-183 delivery attenuates murine lupus nephritis-related injuries via targeting mTOR. Scand J Immunol. 2019;90(5):e12810. doi:10.1111/sji.12810

191. Huang J, Xu X, Wang X, Yang J, Xue M, Yang Y, et al. MicroRNA-590-3p inhibits T helper 17 cells and ameliorates inflammation in lupus mice. Immunology. 2022;165(2):260-73. doi:10.1111/imm.13434

192. Liu R, Ma X, Chen L, Yang Y, Zeng Y, Gao J, et al. MicroRNA-15b Suppresses Th17 Differentiation and Is Associated with Pathogenesis of Multiple Sclerosis by Targeting O-GlcNAc Transferase. J Immunol. 2017;198(7):2626-39. doi:10.4049/jimmunol.1601727

193. Guan D, Li Y, Cui Y, Guo Y, Dong N, Li G, et al. Down-regulated miR-374c and Hsp70 promote Th17 cell differentiation by inducing Fas expression in experimental autoimmune encephalomyelitis. Int J Biol Macromol. 2020;154:1158-65. doi:10.1016/j.ijbiomac.2019.11.147

194. Wang W, Li Y, Fan J, Qu X, Shang D, Qin Q, et al. MiR-365-3p is a negative regulator in IL-17-mediated asthmatic inflammation. Front Immunol. 2022;13:953714. doi:10.3389/fimmu.2022.953714

195. Serr I, Furst RW, Ott VB, Scherm MG, Nikolaev A, Gokmen F, et al. miRNA92a targets KLF2 and the phosphatase PTEN signaling to promote human T follicular helper precursors in T1D islet autoimmunity. Proc Natl Acad Sci U S A. 2016;113(43):E6659-E68. doi:10.1073/pnas.1606646113

196. Pratama A, Srivastava M, Williams NJ, Papa I, Lee SK, Dinh XT, et al. MicroRNA-146a regulates ICOS-ICOSL signalling to limit accumulation of T follicular helper cells and germinal centres. Nat Commun. 2015;6:6436. doi:10.1038/ncomms7436

197. Kang SG, Liu WH, Lu P, Jin HY, Lim HW, Shepherd J, et al. MicroRNAs of the miR-17 approximately 92 family are critical regulators of T(FH) differentiation. Nat Immunol. 2013;14(8):849-57. doi:10.1038/ni.2648

198. Cho S, Lee HM, Yu IS, Choi YS, Huang HY, Hashemifar SS, et al. Differential cell-intrinsic regulations of germinal center B and T cells by miR-146a and miR-146b. Nat Commun. 2018;9(1):2757. doi:10.1038/s41467-018-05196-3

199. Xu X, Li Y, Liang Y, Yin M, Yu Z, Zhang Y, et al. MiR-18a and miR-17 are positively correlated with circulating PD-1(+)ICOS(+) follicular helper T cells after hepatitis B vaccination in a chinese population. BMC Immunol. 2018;19(1):25. doi:10.1186/s12865-018-0263-y

200. Lone W, Bouska A, Sharma S, Amador C, Saumyaranjan M, Herek TA, et al. Genome-Wide miRNA Expression Profiling of Molecular Subgroups of Peripheral T-cell Lymphoma. Clin Cancer Res. 2021;27(21):6039-53. doi:10.1158/1078-0432.CCR-21-0573

201. Liu WH, Kang SG, Huang Z, Wu CJ, Jin HY, Maine CJ, et al. A miR-155-Peli1-c-Rel pathway controls the generation and function of T follicular helper cells. J Exp Med. 2016;213(9):1901-19. doi:10.1084/jem.20160204

202. Liu L, Wang ZG, Pang XL, Feng YH, Wang JX, Xie HC, et al. Bortezomib ameliorates acute allograft rejection after renal transplant by inhibiting Tfh cell proliferation and differentiation via miR-15b/IRF4 axis. Int Immunopharmacol. 2019;75:105758. doi:10.1016/j.intimp.2019.105758

203. Li D, Li X, Duan M, Dou Y, Feng Y, Nan N, et al. MiR-153-3p induces immune dysregulation by inhibiting PELI1 expression in umbilical cord-derived mesenchymal stem cells in patients with systemic lupus erythematosus. Autoimmunity. 2020;53(4):201-9. doi:10.1080/08916934.2020.1750011

204. Liu Y, Wang X, Luan W, Zou J, Xing J, Wang S, et al. MiR-29a-3p negatively regulates circulating Tfh memory cells in patients with Graves' disease by targeting ICOS. Immunol Res. 2023;71(2):173-84. doi:10.1007/s12026-022-09333-5

205. Gao X, Song Y, Du P, Yang S, Cui H, Lu S, et al. Administration of a microRNA-21 inhibitor improves the lupus-like phenotype in MRL/lpr mice by repressing Tfh cell-mediated autoimmune responses. Int Immunopharmacol. 2022;106:108578. doi:10.1016/j.intimp.2022.108578

206. Wu CJ, Cho S, Huang HY, Lu CH, Russ J, Cruz LO, et al. MiR-23~27~24-mediated control of humoral immunity reveals a TOX-driven regulatory circuit in follicular helper T cell differentiation. Sci Adv. 2019;5(12):eaaw1715. doi:10.1126/sciadv.aaw1715

207. Zhang D, Wu Y, Sun G. miR-192 suppresses T follicular helper cell differentiation by targeting CXCR5 in childhood asthma. Scand J Clin Lab Invest. 2018;78(3):236-42. doi:10.1080/00365513.2018.1440628

208. Geng L, Tang X, Zhou K, Wang D, Wang S, Yao G, et al. MicroRNA-663 induces immune dysregulation by inhibiting TGF-beta1 production in bone marrow-derived mesenchymal stem cells in patients with systemic lupus erythematosus. Cell Mol Immunol. 2019;16(3):260-74. doi:10.1038/cmi.2018.1

209. Ripamonti A, Provasi E, Lorenzo M, De Simone M, Ranzani V, Vangelisti S, et al. Repression of miR-31 by BCL6 stabilizes the helper function of human follicular helper T cells. Proc Natl Acad Sci U S A. 2017;114(48):12797-802. doi:10.1073/pnas.1705364114

210. Wang M, Chen H, Qiu J, Yang HX, Zhang CY, Fei YY, et al. Antagonizing miR-7 suppresses B cell hyperresponsiveness and inhibits lupus development. J Autoimmun. 2020;109:102440. doi:10.1016/j.jaut.2020.102440

211. Chen J, Tian J, Tang X, Rui K, Ma J, Mao C, et al. MiR-346 regulates CD4(+)CXCR5(+) T cells in the pathogenesis of Graves' disease. Endocrine. 2015;49(3):752-60. doi:10.1007/s12020-015-0546-5

212. Li X, Sun L, Chen L, Xu Y, Kong X. Upregulation of microRNA-219-5p relieves ulcerative colitis through balancing the differentiation of Treg/Th17 cells. Eur J Gastroenterol Hepatol. 2020;32(7):813-20. doi:10.1097/MEG.0000000000001712

213. Zhao Y, Zheng X, Li M, Zhao J, Wang X, Zhu H. ADAR1 improved Treg cell function through the miR-21b/Foxp3 axis and inhibits the progression of acute graft-versus-host disease after allogeneic hematopoietic stem cell transplantation. Int Immunopharmacol. 2023;115:109620. doi:10.1016/j.intimp.2022.109620

214. Wang J, Zhai X, Guo J, Li Y, Yang Y, Wang L, et al. Long non-coding RNA DQ786243 modulates the induction and function of CD4(+) Treg cells through Foxp3-miR-146a-NF-kappaB axis: Implications for alleviating oral lichen planus. Int Immunopharmacol. 2019;75:105761. doi:10.1016/j.intimp.2019.105761

215. Jin LW, Ye HY, Xu XY, Zheng Y, Chen Y. MiR-133a/133b inhibits Treg differentiation in IgA nephropathy through targeting FOXP3. Biomed Pharmacother. 2018;101:195-200. doi:10.1016/j.biopha.2018.02.022

216. Lu LF, Thai TH, Calado DP, Chaudhry A, Kubo M, Tanaka K, et al. Foxp3-dependent microRNA155 confers competitive fitness to regulatory T cells by targeting SOCS1 protein. Immunity. 2009;30(1):80-91. doi:10.1016/j.immuni.2008.11.010

217. Long CM, Lukomska E, Marshall NB, Nayak A, Anderson SE. Potential Inhibitory Influence of miRNA 210 on Regulatory T Cells during Epicutaneous Chemical Sensitization. Genes (Basel). 2016;8(1). doi:10.3390/genes8010009

218. Zhang Y, Yang Y, Guo J, Cui L, Yang L, Li Y, et al. miR-146a enhances regulatory T-cell differentiation and function in allergic rhinitis by targeting STAT5b. Allergy. 2022;77(2):550-8. doi:10.1111/all.15163

219. Chen Y, Li Z, Liang J, Liu J, Hao J, Wan Q, et al. CircRNA has_circ_0069313 induced OSCC immunity escape by miR-325-3p-Foxp3 axes in both OSCC cells and Treg cells. Aging (Albany NY). 2022;14(10):4376-89. doi:10.18632/aging.204068

220. Liu X, Robinson SN, Setoyama T, Tung SS, D'Abundo L, Shah MY, et al. FOXP3 is a direct target of miR15a/16 in umbilical cord blood regulatory T cells. Bone Marrow Transplant. 2014;49(6):793-9. doi:10.1038/bmt.2014.57

221. Tu J, Zheng N, Mao C, Liu S, Zhang H, Sun L. UC-BSCs Exosomes Regulate Th17/Treg Balance in Patients with Systemic Lupus Erythematosus via miR-19b/KLF13. Cells. 2022;11(24). doi:10.3390/cells11244123

222. Rouas R, Fayyad-Kazan H, El Zein N, Lewalle P, Rothe F, Simion A, et al. Human natural Treg microRNA signature: role of microRNA-31 and microRNA-21 in FOXP3 expression. Eur J Immunol. 2009;39(6):1608-18. doi:10.1002/eji.200838509

223. Yin Y, Cai X, Chen X, Liang H, Zhang Y, Li J, et al. Tumor-secreted miR-214 induces regulatory T cells: a major link between immune evasion and tumor growth. Cell Res. 2014;24(10):1164-80. doi:10.1038/cr.2014.121

224. Yang HY, Barbi J, Wu CY, Zheng Y, Vignali PD, Wu X, et al. MicroRNA-17 Modulates Regulatory T Cell Function by Targeting Co-regulators of the Foxp3 Transcription Factor. Immunity. 2016;45(1):83-93. doi:10.1016/j.immuni.2016.06.022

225. Zhao M, Wang LT, Liang GP, Zhang P, Deng XJ, Tang Q, et al. Up-regulation of microRNA-210 induces immune dysfunction via targeting FOXP3 in CD4(+) T cells of psoriasis vulgaris. Clin Immunol. 2014;150(1):22-30. doi:10.1016/j.clim.2013.10.009

226. Huang Z, Liu F, Wang W, Ouyang S, Sang T, Huang Z, et al. Deregulation of circ_003912 contributes to pathogenesis of erosive oral lichen planus by via sponging microRNA-123, -647 and -31 and upregulating FOXP3. Mol Med. 2021;27(1):132. doi:10.1186/s10020-021-00382-4

227. Lyszkiewicz M, Winter SJ, Witzlau K, Fohse L, Brownlie R, Puchalka J, et al. miR-181a/b-1 controls thymic selection of Treg cells and tunes their suppressive capacity. PLoS Biol. 2019;17(3):e2006716. doi:10.1371/journal.pbio.2006716

228. Lu LF, Boldin MP, Chaudhry A, Lin LL, Taganov KD, Hanada T, et al. Function of miR-146a in controlling Treg cell-mediated regulation of Th1 responses. Cell. 2010;142(6):914-29. doi:10.1016/j.cell.2010.08.012

229. Zheng Y, Dong C, Yang J, Jin Y, Zheng W, Zhou Q, et al. Exosomal microRNA-155-5p from PDLSCs regulated Th17/Treg balance by targeting sirtuin-1 in chronic periodontitis. J Cell Physiol. 2019;234(11):20662-74. doi:10.1002/jcp.28671

230. Xu M, Liu Q, Li S, Zhang W, Huang X, Han K, et al. Increased expression of miR-338-3p impairs Treg-mediated immunosuppression in pemphigus vulgaris by targeting RUNX1. Exp Dermatol. 2020;29(7):623-9. doi:10.1111/exd.14111

231. He R, Chen Y, Chen X, Yuan B. Mechanism of miR-181a-5p in Regulatory T/T-Helper 17 Immune Imbalance and Asthma Development in Mice with Allergic Rhinitis. Int Arch Allergy Immunol. 2022;183(4):375-88. doi:10.1159/000519703

232. Zhu F, Li H, Liu Y, Tan C, Liu X, Fan H, et al. miR-155 antagomir protect against DSS-induced colitis in mice through regulating Th17/Treg cell balance by Jarid2/Wnt/beta-catenin. Biomed Pharmacother. 2020;126:109909. doi:10.1016/j.biopha.2020.109909

233. Geng X, Mao G, Zhao D, Xiang Y, Wang M, Yu G, et al. Downregulation of miR-33a/b and miR-181a contributes to recurrent pregnancy loss by upregulating S1PR1 and repressing regulatory T cell differentiation. Placenta. 2022;121:137-44. doi:10.1016/j.placenta.2022.03.011

234. Wang Y, Tu S, Huang Y, Qin K, Chen Z. MicroRNA-181a regulates Treg functions via TGF-beta1/Smad axis in the spleen of mice with acute gouty arthritis induced by MSU crystals. Braz J Med Biol Res. 2022;55:e12002. doi:10.1590/1414-431X2022e12002

235. Zhang D, Qiu X, Li J, Zheng S, Li L, Zhao H. MiR-23a-3p-regulated abnormal acetylation of FOXP3 induces regulatory T cell function defect in Graves' disease. Biol Chem. 2019;400(5):639-50. doi:10.1515/hsz-2018-0343

236. Scherm MG, Serr I, Zahm AM, Schug J, Bellusci S, Manfredini R, et al. miRNA142-3p targets Tet2 and impairs Treg differentiation and stability in models of type 1 diabetes. Nat Commun. 2019;10(1):5697. doi:10.1038/s41467-019-13587-3

237. Wang Y, Xie C, Song Y, Xiang W, Peng J, Han L, et al. miR-20a suppresses Treg differentiation by targeting Map3k9 in experimental autoimmune encephalomyelitis. J Transl Med. 2021;19(1):223. doi:10.1186/s12967-021-02893-4

238. Chen J, Zhang Y, Tan W, Gao H, Xiao S, Gao J, et al. Silencing of long non-coding RNA NEAT1 improves Treg/Th17 imbalance in preeclampsia via the miR-485-5p/AIM2 axis. Bioengineered. 2021;12(1):8768-77. doi:10.1080/21655979.2021.1982306

239. Kim D, Nguyen QT, Lee J, Lee SH, Janocha A, Kim S, et al. Anti-inflammatory Roles of Glucocorticoids Are Mediated by Foxp3(+) Regulatory T Cells via a miR-342-Dependent Mechanism. Immunity. 2020;53(3):581-96 e5. doi:10.1016/j.immuni.2020.07.002

240. Warth SC, Hoefig KP, Hiekel A, Schallenberg S, Jovanovic K, Klein L, et al. Induced miR-99a expression represses Mtor cooperatively with miR-150 to promote regulatory T-cell differentiation. EMBO J. 2015;34(9):1195-213. doi:10.15252/embj.201489589

241. Rodriguez-Munoz A, Martinez-Hernandez R, Ramos-Levi AM, Serrano-Somavilla A, Gonzalez-Amaro R, Sanchez-Madrid F, et al. Circulating Microvesicles Regulate Treg and Th17 Differentiation in Human Autoimmune Thyroid Disorders. J Clin Endocrinol Metab. 2015;100(12):E1531-9. doi:10.1210/jc.2015-3146

242. Singh Y, Garden OA, Lang F, Cobb BS. MicroRNA-15b/16 Enhances the Induction of Regulatory T Cells by Regulating the Expression of Rictor and mTOR. J Immunol. 2015;195(12):5667-77. doi:10.4049/jimmunol.1401875

243. Liu Y, Li C, Yang Y, Li T, Xu Y, Zhang W, et al. The TGF-beta/miR-31/CEACAM1-S axis inhibits CD4(+) CD25(+) Treg differentiation in systemic lupus erythematosus. Immunol Cell Biol. 2021;99(7):697-710. doi:10.1111/imcb.12449

244. Kimura K, Hohjoh H, Fukuoka M, Sato W, Oki S, Tomi C, et al. Circulating exosomes suppress the induction of regulatory T cells via let-7i in multiple sclerosis. Nat Commun. 2018;9(1):17. doi:10.1038/s41467-017-02406-2

245. Pang XL, Wang ZG, Liu L, Feng YH, Wang JX, Xie HC, et al. Immature dendritic cells derived exosomes promotes immune tolerance by regulating T cell differentiation in renal transplantation. Aging (Albany NY). 2019;11(20):8911-24. doi:10.18632/aging.102346

246. Becker W, Nagarkatti M, Nagarkatti PS. miR-466a Targeting of TGF-beta2 Contributes to FoxP3(+) Regulatory T Cell Differentiation in a Murine Model of Allogeneic Transplantation. Front Immunol. 2018;9:688. doi:10.3389/fimmu.2018.00688

247. Bronevetsky Y, Burt TD, McCune JM. Lin28b Regulates Fetal Regulatory T Cell Differentiation through Modulation of TGF-beta Signaling. J Immunol. 2016;197(11):4344-50. doi:10.4049/jimmunol.1601070

248. Lv Q, Shi C, Qiao S, Cao N, Guan C, Dai Y, et al. Alpinetin exerts anti-colitis efficacy by activating AhR, regulating miR-302/DNMT-1/CREB signals, and therefore promoting Treg differentiation. Cell Death Dis. 2018;9(9):890. doi:10.1038/s41419-018-0814-4

249. Zhang L, Ke F, Liu Z, Bai J, Liu J, Yan S, et al. MicroRNA-31 negatively regulates peripherally derived regulatory T-cell generation by repressing retinoic acid-inducible protein 3. Nat Commun. 2015;6:7639. doi:10.1038/ncomms8639

250. Li X, Cai W, Xi W, Sun W, Shen W, Wei T, et al. MicroRNA-31 Regulates Immunosuppression in Ang II (Angiotensin II)-induced Hypertension by Targeting Ppp6C (Protein Phosphatase 6c). Hypertension. 2019;73(5):e14-e24. doi:10.1161/HYPERTENSIONAHA.118.12319

251. Pei X, Wang X, Li H. LncRNA SNHG1 regulates the differentiation of Treg cells and affects the immune escape of breast cancer via regulating miR-448/IDO. Int J Biol Macromol. 2018;118(Pt A):24-30. doi:10.1016/j.ijbiomac.2018.06.033

252. Wang WL, Ouyang C, Graham NM, Zhang Y, Cassady K, Reyes EY, et al. microRNA-142 guards against autoimmunity by controlling Treg cell homeostasis and function. PLoS Biol. 2022;20(2):e3001552. doi:10.1371/journal.pbio.3001552

253. Kelada S, Sethupathy P, Okoye IS, Kistasis E, Czieso S, White SD, et al. miR-182 and miR-10a are key regulators of Treg specialisation and stability during Schistosome and Leishmania-associated inflammation. PLoS Pathog. 2013;9(6):e1003451. doi:10.1371/journal.ppat.1003451

254. Song J, Lin Z, Liu Q, Huang S, Han L, Fang Y, et al. MiR-192-5p/RB1/NF-kappaBp65 signaling axis promotes IL-10 secretion during gastric cancer EMT to induce Treg cell differentiation in the tumour microenvironment. Clin Transl Med. 2022;12(8):e992. doi:10.1002/ctm2.992

255. Liu N, Chang CW, Steer CJ, Wang XW, Song G. MicroRNA-15a/16-1 Prevents Hepatocellular Carcinoma by Disrupting the Communication Between Kupffer Cells and Regulatory T Cells. Gastroenterology. 2022;162(2):575-89. doi:10.1053/j.gastro.2021.10.015

256. Wei Y, Jing J, Peng Z, Liu X, Wang X. Acacetin ameliorates insulin resistance in obesity mice through regulating Treg/Th17 balance via MiR-23b-3p/NEU1 Axis. BMC Endocr Disord. 2021;21(1):57. doi:10.1186/s12902-021-00688-8

257. de Kouchkovsky D, Esensten JH, Rosenthal WL, Morar MM, Bluestone JA, Jeker LT. microRNA-17-92 regulates IL-10 production by regulatory T cells and control of experimental autoimmune encephalomyelitis. J Immunol. 2013;191(4):1594-605. doi:10.4049/jimmunol.1203567

258. Li P, Wang J, Guo F, Zheng B, Zhang X. A novel inhibitory role of microRNA-224 in particulate matter 2.5-induced asthmatic mice by inhibiting TLR2. J Cell Mol Med. 2020;24(5):3040-52. doi:10.1111/jcmm.14940

259. Ning T, Li J, He Y, Zhang H, Wang X, Deng T, et al. Exosomal miR-208b related with oxaliplatin resistance promotes Treg expansion in colorectal cancer. Mol Ther. 2021;29(9):2723-36. doi:10.1016/j.ymthe.2021.04.028

260. Li W, Kong LB, Li JT, Guo ZY, Xue Q, Yang T, et al. MiR-568 inhibits the activation and function of CD4(+) T cells and Treg cells by targeting NFAT5. Int Immunol. 2014;26(5):269-81. doi:10.1093/intimm/dxt065

261. Khosravi M, Karimi MH, Hossein Aghdaie M, Kalani M, Naserian S, Bidmeshkipour A. Mesenchymal stem cells can induce regulatory T cells via modulating miR-126a but not miR-10a. Gene. 2017;627:327-36. doi:10.1016/j.gene.2017.06.012

262. Li JQ, Hu SY, Wang ZY, Lin J, Jian S, Dong YC, et al. Long non-coding RNA MEG3 inhibits microRNA-125a-5p expression and induces immune imbalance of Treg/Th17 in immune thrombocytopenic purpura. Biomed Pharmacother. 2016;83:905-11. doi:10.1016/j.biopha.2016.07.057

263. Jeker LT, Zhou X, Blelloch R, Bluestone JA. DGCR8-mediated production of canonical microRNAs is critical for regulatory T cell function and stability. PLoS One. 2013;8(5):e66282. doi:10.1371/journal.pone.0066282

264. Shao MM, Pei XB, Chen QY, Wang F, Wang Z, Zhai K. Macrophage-derived exosome promotes regulatory T cell differentiation in malignant pleural effusion. Front Immunol. 2023;14:1161375. doi:10.3389/fimmu.2023.1161375

265. Fang T, Li J, Wu X. Shenmai injection improves the postoperative immune function of papillary thyroid carcinoma patients by inhibiting differentiation into Treg cells via miR-103/GPER1 axis. Drug Dev Res. 2018;79(7):324-31. doi:10.1002/ddr.21459

266. Zhou H, Lou F, Bai J, Sun Y, Cai W, Sun L, et al. A peptide encoded by pri-miRNA-31 represses autoimmunity by promoting T(reg) differentiation. EMBO Rep. 2022;23(5):e53475. doi:10.15252/embr.202153475

267. Cui B, Chen XJ, Sun J, Li SP, Zhou GP, Sun LY, et al. Dendritic cells originating exosomal miR-193b-3p induces regulatory T cells to alleviate liver transplant rejection. Int Immunopharmacol. 2023;114:109541. doi:10.1016/j.intimp.2022.109541

268. Yin X, Ge J, Ge X, Gao J, Su X, Wang X, et al. MiR-363-5p modulates regulatory T cells through STAT4-HSPB1-Notch1 axis and is associated with the immunological abnormality in Graves' disease. J Cell Mol Med. 2021;25(19):9364-77. doi:10.1111/jcmm.16876

269. Ren JP, Wang L, Zhao J, Wang L, Ning SB, El Gazzar M, et al. Decline of miR-124 in myeloid cells promotes regulatory T-cell development in hepatitis C virus infection. Immunology. 2017;150(2):213-20. doi:10.1111/imm.12680

270. Zhong C, Tao B, Li X, Xiang W, Peng L, Peng T, et al. HOXA-AS2 contributes to regulatory T cell proliferation and immune tolerance in glioma through the miR-302a/KDM2A/JAG1 axis. Cell Death Dis. 2022;13(2):160. doi:10.1038/s41419-021-04471-4

271. Xu YJ, Zhao JM, Gao C, Ni XF, Wang W, Hu WW, et al. Hsa_circ_0136666 activates Treg-mediated immune escape of colorectal cancer via miR-497/PD-L1 pathway. Cell Signal. 2021;86:110095. doi:10.1016/j.cellsig.2021.110095

272. Yu WQ, Ji NF, Gu CJ, Sun ZX, Wang ZX, Chen ZQ, et al. Downregulation of miR-4772-3p promotes enhanced regulatory T cell capacity in malignant pleural effusion by elevating Helios levels. Chin Med J (Engl). 2019;132(22):2705-15. doi:10.1097/CM9.0000000000000517

273. Ye SB, Zhang H, Cai TT, Liu YN, Ni JJ, He J, et al. Exosomal miR-24-3p impedes T-cell function by targeting FGF11 and serves as a potential prognostic biomarker for nasopharyngeal carcinoma. J Pathol. 2016;240(3):329-40. doi:10.1002/path.4781
